# Conflict, mental health, and labor productivity: evidence from hired farm workers in Myanmar

**DOI:** 10.1186/s44263-026-00307-5

**Published:** 2026-07-29

**Authors:** Andrew Laitha, Anna Fabry, Bart Minten, Eva-Marie Meemken

**Affiliations:** 1 Food Systems Economics and Policy Group – ETH Zurich, Zurich, Switzerland; 2 International Food Policy Research Institute (IFPRI), Yangon, Myanmar

**Keywords:** Conflict, Hired farm workers, Mental health, Trauma, Labor productivity, Absenteeism, Work performance

## Abstract

**Background:**

Violent conflict can affect worker’s mental health and labor productivity, with implications for agricultural production and food security. Hired farm workers are particularly vulnerable because they often rely on daily wages, have limited assets, and face insecure employment, yet evidence on how conflict affects their mental health and productivity remains limited. This study examines the associations between conflict exposure, mental health, and labor productivity among hired farm workers in Myanmar.

**Methods:**

We conducted a cross-sectional phone survey of 1,504 hired farm workers across Myanmar. The survey collected information on workers’ demographic characteristics, work activities during the past 12 months, absenteeism, self-reported labor productivity (presenteeism), agricultural labor market conditions, job characteristics, job and life satisfaction, mental health, workplace harassment, and access to information. These primary survey data were matched with conflict event data from the Armed Conflict Location & Event Data (ACLED). Mediation analysis was used to examine whether mental health mediates the relationship between conflict exposure and labor productivity outcomes.

**Results:**

Overall, 39% of workers were exposed to conflict and 20% met the criteria for trauma. Compared with non-exposed workers, conflict-exposed workers were more likely to experience trauma (27% vs. 17%), more frequently absent from work (28% vs. 23%), and reported lower work performance (7.0 vs. 7.4). In multivariable mediation analyses, conflict exposure was associated with a 7-percentage-point higher likelihood of absenteeism and a 0.15-point lower work performance score. Trauma significantly mediated these associations, increasing the total association between conflict exposure and absenteeism by approximately 18%.

**Conclusions:**

Our findings highlight the need for greater research and policy attention to the socio-psychological implications of conflict, which can erode labor productivity. They also reinforce the case for increased investment in mental health interventions, which have been severely reduced amid widespread budget cuts despite their potentially high cost-effectiveness.

**Supplementary Information:**

The online version contains supplementary material available at 10.1186/s44263-026-00307-5.

## Background

The world is experiencing an unprecedented rise in violent conflict [1], with devastating effects on multiple Sustainable Development Goals (SDGs), such as poverty reduction (SDG 1), zero hunger (SDG 2), mental health (SDG 3), and gender equality (SDG 5). While a growing body of literature documents the negative implications of conflict on food security and livelihoods [2–5] the mechanisms through which conflict undermines welfare have received insufficient attention. This paper contributes novel evidence by focusing on the links between conflict, mental health, and labor productivity—thereby bridging three bodies of literature and disciplinary perspectives.

The first body of literature, mostly in psychology, examines the first link, namely between conflict and mental health [6–10]. This literature finds that conflict is associated with increased mental health problems, including post-traumatic stress disorder (PTSD), depression, and anxiety [6, 7, 11]. This literature has typically focused on specific populations such as internally displaced persons or refugees [10, 12, 13]. Most related to our farm and labor focus are recent studies examining the relationship between exposure to shocks (such as violence and crop price volatility) and poor mental health among farmers [14–17]. This literature has mostly overlooked the development-relevant consequences of deteriorating mental health.

The second body of literature, concentrated in development economics, focuses on these socio-economic effects of conflict [4, 18–20]—while mostly overlooking mental health as a mechanism, with a few notable exceptions [8, 18, 21–24]. Most of the latter studies focus on poverty, income, and food insecurity as the key outcomes, with so far limited attention to labor productivity, despite the key importance of labor for food security and livelihoods. The focus on labor as a pathway connects to the third body of literature.

The third body of literature, concentrated in economics and organizational psychology, focuses on the second link, namely mental health and labor productivity [22, 25–28]. This literature focuses on higher-income countries and tests the ‘happy-productive worker’ viewpoint, which posits that worker well-being is a positive determinant of labor and firm productivity growth [29, 30]. Confirming this proposition, several studies find that poor mental health is associated with (i) increased absenteeism, i.e., the failure to report for scheduled work, and (ii) reduced work performance, i.e., attending work with reduced job performance [25, 26, 31, 32]. However, these studies focus on higher-income countries and do not consider conflict as the cause of poor mental health [33–35].

This paper bridges these three bodies of literature by examining the link between conflict, mental health, and labor productivity—a relationship that, to our knowledge, has not been studied. Moreover, we contribute by focusing on a critically important yet understudied group: hired farm workers. Farm labor is an essential input to sustain food production, food security, and livelihoods [36], particularly in conflict-affected regions where basic needs, such as food, are most at risk [4, 5, 37, 38]. Furthermore, the farm workforce represents a huge group as agriculture employs one-fourth of the global workforce [39], around 40% of them as hired workers [40]. Lastly, we contribute by integrating a gendered lens, highlighting the gendered patterns of agricultural labor [41, 42] and that conflict, often accompanied by gender-based violence, differentially impacts women and men [43–45].

We collected data from hired farm workers (*N* = 1,504) through a nation-wide survey across Myanmar between 29th November and 20th December 2023. Myanmar is a suitable case study where our research focus is highly relevant. Following the 2021 military coup, Myanmar has faced a severe humanitarian crisis, with 19.9 million people requiring aid and 3.5 million internally displaced [46].

## Methods

### Study setting

Myanmar has experienced recurrent armed conflict since gaining independence in 1948, with violence intensifying following the military coup in February 2021 and the subsequent armed resistance [47, 48]. This study was conducted in this conflict-affected setting using a nationwide survey of hired farm workers. Hired farm workers are primarily employed as daily wage laborers in crop production, undertaking activities such as land preparation, planting, weeding, agrochemical application, irrigation, harvesting, and post-harvest crop handling. Most work in the production of paddy, pulses, and maize. Hired farm workers account for approximately 11% of Myanmar’s population and are predominantly located in rural areas [49, 50].

### Survey and data

We use primary survey data from hired farm workers, which we collected via computer-assisted telephone interviews (CATI) between 29th November and 20th December 2023 using the SurveyToGo platform (Dooblo Ltd., Kfar Saba, Israel). Phone surveys have been widely used since the pandemic, when curfews and movement restrictions hindered face-to-face interviews [51–54]. Phone surveys offer similar advantages in conflict-affected Myanmar, where face-to-face surveys struggle to reach many locations due to conflict, related security issues, or geographical isolation [55]. An additional advantage of phone surveys is that social desirability bias may be less of an issue in phone surveys because the absence of face-to-face interaction reduces the pressure to give socially acceptable responses. Without visual cues or the physical presence of an interviewer, respondents often feel more anonymous and comfortable disclosing sensitive information.

To further improve data quality, we followed a detailed protocol and worked with experienced enumerators who have been conducting CATI interviews for various phone surveys in Myanmar, including with the World Bank, the United Nations Development Program (UNDP), and the International Food Policy Research Institute (IFPRI).

Our target population for the survey consists of farm workers, i.e., individuals whose households’ main income come from hired farm labor. To find and sample this population, we drew on an existing, large household survey, the Myanmar Household Welfare Survey (MHWS) [56]. Specifically, we reinterviewed farm workers who participated in the MHWS, using detailed questionnaire modules on labor and mental health (see below), which are absent from the MHWS questionnaire. This strategy yields detailed and rare national-wide farm worker data amidst conflict.

The study team included researchers with regional expertise and contextual familiarity; in particular, the first author (Dr. Andrew Laitha) is of Myanmar origin, and co-author Dr. Bart Minten is based in the region (Myanmar/Laos), which supported data collection design and interpretation.

As we leverage the MHWS for sampling, we briefly describe its original sampling strategy, which was designed to maximize both response rate and representativeness. For this purpose, Myanmar Survey Research (MSR) selected respondents from an existing database of 280,274 phone numbers of adults who had consented to participate in phone-based interviews. This database was compiled using a combination of random digit dialing and participant referrals [56]. To construct a nationally representative sample, MSR developed a master list in which all phone numbers were stratified by township, with the number of entries per township proportional to its population size according to the 2014 Census [56]. Within each township, households were randomly selected for contact. This township-level stratification was intended to minimize bias toward wealthier or more connected communities. Additional quotas were also applied by state or region to maintain balanced representation by gender (50% women), rural residence, agricultural livelihood, and education level, thereby reducing the risk of underrepresentation among these groups [56]. We followed the same sampling strategy for hired farm worker survey.

We conducted our phone survey among 1,504 hired farm workers in Myanmar. The hired farm worker survey instrument was developed and tested after conducting 20 qualitative interviews with both hired farm workers and farmers in Myanmar. The qualitative interviews were conducted by the first author (Dr. Andrew Laitha) using telephone calls, Viber calls, and Facebook Messenger. A semi-structured approach was used rather than a fully standardized interview guide. Respondents were asked open-ended questions on their daily livelihoods, wage levels, and how hired farm workers access employment opportunities. Farmers were asked about how they recruit hired labor and how labor arrangements and contracts have evolved in recent years. The survey questionnaire captures information such as respondent demographic information, farm and off-farm activities in the past 12 months, self-reported labor productivity, agricultural labor market conditions before and after the military coup, job characteristics, job satisfaction, life satisfaction, trauma, mental distress (anxiety and depression), workplace harassment, and access to information.

All survey modules were translated into Burmese, the official language of Myanmar (English version can be found in Supplementary Material 1). Translated versions of the mental health-related modules were carefully reviewed by a clinical psychologist from the Mental Health Psychosocial Support (MHPSS) Working Group in Myanmar. Enumerators were equipped with culturally sensitive interview techniques and trained in basic counselling skills to assist respondents in coping with and overcoming distress. They also received training on how to respond to secondary trauma, including safety protocols such as when to pause or stop the interview and provide basic counselling if respondents exhibited elevated levels of depression or suicidal ideation. Enumerators shared the contact information of mental health counsellors with respondents who agreed to receive it.

### Secondary conflict data

In addition to our primary data, we use secondary conflict data from the Armed Conflict Location & Event Data (ACLED) [57]. ACLED provides geo-referenced information on conflict events and conflict-related fatalities from 2010 onward [57]. The dataset includes a wide range of event types, such as battles, explosive or remote violence, violence against civilians, protests, riots, and strategic developments. These events are coded based on reports from news outlets. Alternative datasets, such as the Uppsala Conflict Data Program’s Georeferenced Event Dataset (UCDP GED), capture conflict events at larger administrative units in Myanmar (i.e., states/regions). Thus, we prefer ACLED, which provides more granular event classifications at the township level. None of the commonly-used data sets captures the individual level [58–60]. However, individual level-conflict exposure data comes with its own challenges, e.g., as it is typically self-reported.

We therefore match our primary data to ACLED at the township level. Myanmar has 330 townships, which constitute the third tier of the administrative system. Townships belong to 7 states, 7 regions, and 1 union territory, which together constitute the second level of administration. These 15 administrative units do not overlap (are mutually exclusive).

## Measurement of key variables and analysis method

### Conflict

We proxy conflict using several approaches. In our main specification, we use the Conflict Severity Index, CSI [61, 62]. The CSI is derived from four key indicators, namely deadliness, danger, diffusion, and fragmentation, which are derived from the ACLED data and displayed in Table 1. Unlike simple conflict death counts, the CSI better captures the multifaceted nature of conflict by incorporating spatial spread, persistence, and intensity beyond fatal incidents. This index has been used in previous studies [61, 62]. The CSI ranges from 0 to 4, where 0 indicates no conflict, 1 indicates limited conflict severity, 2 moderate severity, 3 high severity, and 4 extreme severity. The index is aggregated at the township level (Table 1).

**Table 1:** Description of indicators to construct the Conflict Severity Index (CSI)

Indicator	Description	Threshold	CSI
Deadliness	All fatalities (count of all events in a given period)	Mean	0–4
Danger	Count of all events categorized as “violence against civilians” standardized by population density (2020) in a given period	Median	0–4
Diffusion	Share of village tracks (VTs) with higher average weekly event counts in a given period	1·5 weekly average	0–4
Fragmentation	Number of violent armed group actors in a given period, excluding unidentified groups and civilians	> 80 percentile	0–4

*Note*: We built the indicators based on the Armed Conflict Location & Event Data (ACLED) Conflict Severity Index [63], adapted to the Myanmar context [61, 62]

**Fig. 1:**
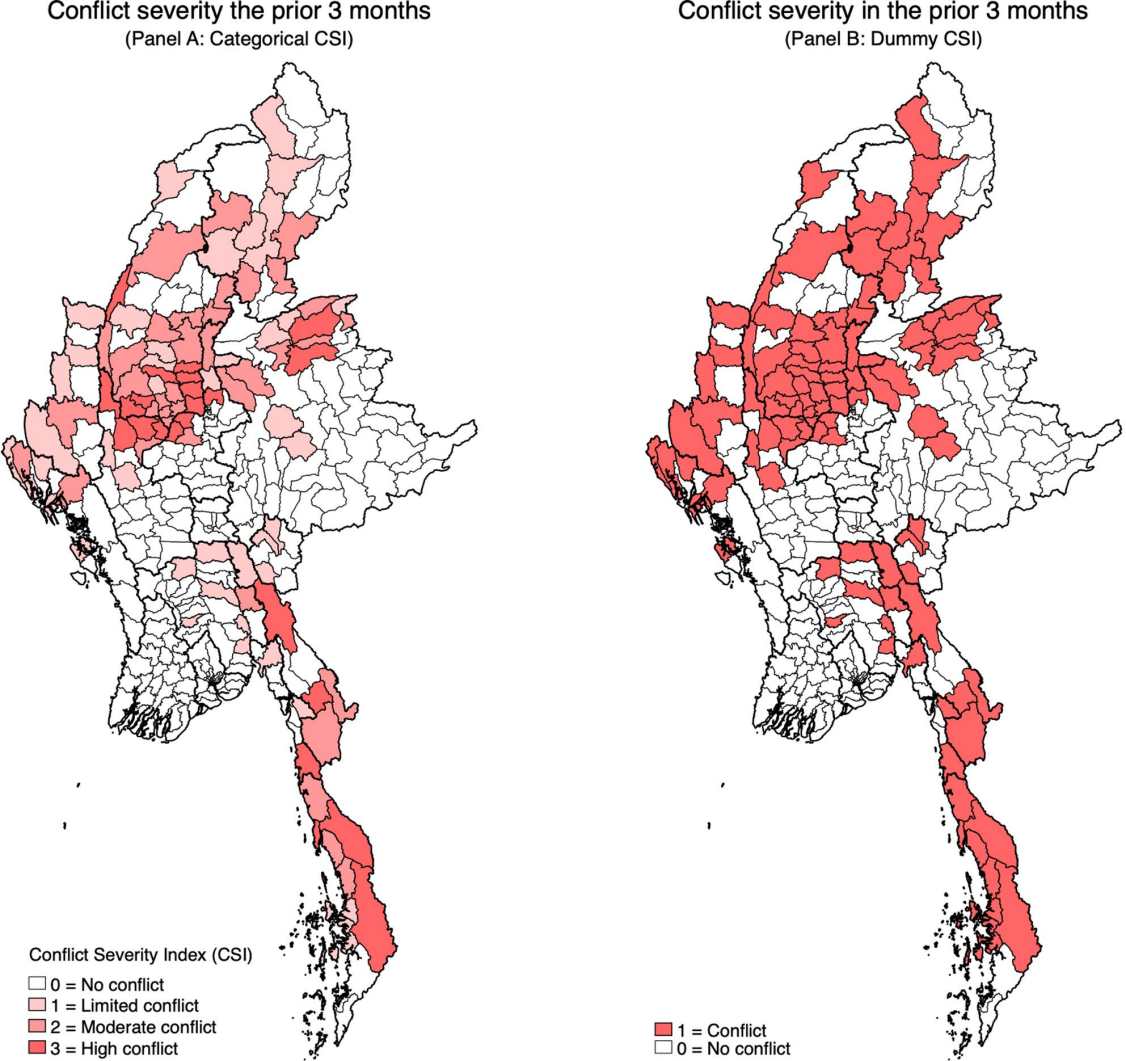
*Note*: Variation of conflict across townships. The CSI timeframe is from 28th August and 28th November 2023. The thin dark lines represent the borders of 330 townships, at which the CSI is measured. The thick dark lines represent the borders of 7 states, 7 regions, and 1 union territory. Panel **A** represents the CSI, which ranges from 0 to 4 (note that there are no observations with CSI = 4, indicating extreme severity of conflict during a 3-month conflict exposure period), while Panel **B** shows the dummy variable indicating either no conflict (CSI = 0) or conflict (CSI = 1–4)

For the purpose of this study, we use a binary conflict measure rather than the categorical CSI, as we want to compare conflict-affected workers with those who are not affected by conflict. This dummy variable equals one if a township experienced any level of conflict (CSI = 1–4) and zero if no conflict events were recorded (CSI = 0). This categorization divides our sample into two broad groups: 61% of respondents are in areas without conflict (CSI = 0), and 39% in areas with conflict (CSI = 1–4). Within the latter group, 14% of respondents live in townships with limited conflict (CSI = 1), 13% in townships with moderate conflict (CSI = 2), and 12% in townships with high conflict (CSI = 3). No townships recorded extreme conflict (CSI = 4) during the study period. Figure 1 illustrates this spatial variation in conflict severity across townships, showing that some townships are affected (highlighted in red) while others are not (highlighted in white). The left panel presents the categorical CSI (0–4), whereas the right panel shows the binary CSI indicator.

To construct the CSI, we capture conflicts that occurred in the monsoon season (28th of August 2023 to 28th of November 2023), directly before data collection started (28th of November). As a robustness check, we also use alternative periods, creating a 6-month and a 12-month CSI. As another robustness check and to add insights on conflict intensity, we use the CSI as a continuous variable (CSI = 0–4). Additionally, we use an alternative conflict measure, namely fatalities.

### Mental health

To measure mental health, we use individual-level data from our farm survey (see above), with a particular focus on trauma. Trauma is an important indicator because it is strongly associated with the risk of developing post-traumatic stress disorder (PTSD), a condition commonly observed among populations exposed to violence, including those affected by armed conflict [6, 7].

To measure trauma, we use a common method, the Trauma Screening Questionnaire (TSQ) [64–66]. TSQ is a validated instrument to identify individuals at elevated risk of PTSD. The TSQ includes 10 items, which assess respondents’ reactions to past traumatic events they experienced, indicating (yes or no) if they experienced any of the following 10 items at least twice. The ten items include upsetting thoughts or memories about the event, upsetting dreams about the event, acting or feeling as though the event were happening again, feeling upset by reminders of the event, bodily reactions when reminded of the event, difficulty falling asleep, outbursts of anger, difficulty concentrating, heightened awareness of potential dangers, and being startled at something unexpected [65].

In our analysis, we treat trauma as a binary variable that takes the value 1 when a respondent’s TSQ score is five or higher, and zero otherwise. This threshold is consistent with those used in recent studies [67, 68]. The internal consistency and reliability are very strong (Cronbach: 0.87) [69] and similar to previous studies, which reported a Cronbach of 0.85 [64, 66]. As an additional robustness check, we also draw on the Hopkins Symptoms Checklist (HSCL-10) –] [70–72] as an alternative measure of mental distress, which is further described in the Supplementary Material 2.

### Labor productivity

For labor productivity, we focus on workers’ absenteeism and work performance. These measures are commonly employed as proxies for labor productivity as they can capture the health-related loss of productivity among workers in various contexts [73–75]. We use the World Health Organization Health and Work Performance Questions (WHO-HPQ) to measure absenteeism and work performance [76], adapting the questionnaire for hired farm workers. We define absenteeism as a dummy variable that equals one if the worker reports having worked fewer hours in the last seven days than committed, and zero otherwise [25, 76]. Workers are generally unable to miss agreed-upon workdays with farm owners, as farming requires adherence to seasonal patterns. To measure work performance, workers were asked to rate their work performance on a 0–10 scale (0 indicating the worst work performance anyone could have at work, and 10 the performance of a top worker) for the days the worker worked during the past seven days [32, 76, 77].

### Analysis

**Fig. 2:**
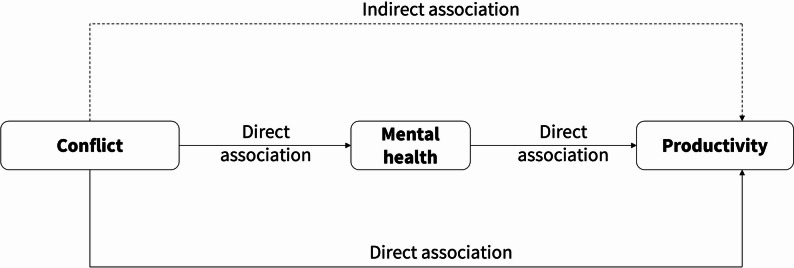
*Note*: The direct and indirect associations between conflict, mental health, and labor productivity. This diagram visually illustrates how conflict may be associated with labor productivity outcomes (i.e., absenteeism and work performance), both directly and through its relationship with mental health (trauma). The arrows indicate the direction of the relationships among the three main variables

To analyze the relationship between conflict, mental health (trauma), and labor productivity (absenteeism and work performance), we use mediation analysis [78, 79], a method widely used among psychologists, social scientists, and beyond [80–82]. Mediation analysis serves to analyze the mechanisms through which one variable is associated with another. This is illustrated in Fig. 2, which shows the pathways considered in our analysis. In this study, we examine the relationship between conflict exposure and labor productivity, and how this relationship operates through trauma as a mediating variable.

We estimate the mediation model using the *“mediate”* command in StataNow/SE 19.5 (StataCorp LLC, College Station, TX, USA) (Supplementary Material 3). Descriptive statistics were used to summarize the data. Statistical significance was assessed using two-sided tests with a significance level of *P* < 0.05. Regression analyses were performed to examine associations between conflict and mental health, and between mental health and labor productivity. Mediation analyses were conducted to assess indirect association between conflict and labor productivity. For absenteeism (a binary variable) and trauma (a binary variable), we estimate probit models. For work performance (a continuous variable), we estimate linear models. We control for observable individual characteristics, including gender (coded as 1 if male, 0 otherwise), age, marital status (1 if married, 0 otherwise), years of work experience, education (1 if the respondent has at least primary education, 0 otherwise), relationship with the employer (1 if relative/friend, 0 otherwise), and whether the household is based in a rural area (1 if rural, 0 if urban).

Additionally, we include location fixed effects for the 15 second-level administrative units (i.e., 7 states, 7 regions, and 1 union territory) to control for broader area characteristics—such as proximity to borders, economic conditions, infrastructure, and geographic factors—that may influence our main variables of interest and are thus useful to control for. However, including location fixed effects requires meaningful within variation. Table S1 in the Supplementary Material 2 shows that the share of townships exposed to conflict within these units (states, regions, or the union territory), varies between 0 and 90% (with an average of 41%). This gives sufficient within-variation to include location fixed effects in our main model specification. However, to explore this further, we also estimate the same models without location fixed effects, compare the results, and conduct additional robustness checks (see results section).

Interpreting mediation results as causal requires several strong assumptions. First, exposure to violent conflict is measured prior to the measurement of trauma and productivity. This temporal ordering reduces the likelihood of a bidirectional relationship between conflict and either trauma or productivity. Trauma and productivity, however, are measured at the same time. We therefore cannot rule out the possibility of a bidirectional relationship between these two variables, although previous research suggests that trauma is related to employment outcomes rather than the reverse [22].

Second, we seek to approximate the sequential ignorability assumption, which would require that (i) conflict exposure is as good as random once observable characteristics are controlled for, and (ii) the likelihood of experiencing trauma is independent of conflict exposure conditional on the same covariates [78]. Sequential ignorability also assumes that no unobserved variables simultaneously influence both trauma and productivity. Because our data are observational rather than experimental, and although we control for a broad set of potential confounders and location fixed effects (see above), we cannot rule out that some assumptions are not fully satisfied. We also ensure that our specification does not introduce a bad control problem, as discussed by Chen et al. [83]. For example, we lack data on individuals’ history of mental health support, which is thus unobserved, though not very common.

Thus, we refrain from making causal claims and interpret results as associations. Because conflict is impossible to randomize for the purpose of generating experimental research data; and as some possibly relevant variables are always unobserved—most related studies share this limitation.

## Results

### Descriptive results

We start with an overview, illustrating the link between conflict, trauma, and productivity descriptively. Table 2 provides an overview of the characteristics of the sampled hired farm workers, for the full sample (Column 1). Columns 2 and 3 compare workers affected by conflict with those who are not, with the corresponding p-values reported in Column 4.

The average age of our sample is 36 years, 39% are men, and the vast majority (93%) reside in rural areas of Myanmar (Table 2, Column 1). Education levels are low—only 28% have completed primary school—and the average work experience is 10 years. The main income of these workers comes from farm work, receiving daily wages, although a few workers (*N* = 145) supplement this income with seasonal off-farm jobs like construction.

Regarding our key variables of interest, 25% of workers reported missing work in the past week, with an average absence of four hours (ranging from 0 to 77). Average work performance is rated at 7 on a scale from 0 (low) to 10 (high). Around 20% of respondents meet the criteria for trauma. In total, 39% of the sample is exposed to conflict. Descriptive statistics in Table 2  (Columns 2–4) further show that conflict-exposed workers display significantly lower productivity than non-exposed workers: conflict-exposed workers are more frequently absent (28% vs. 23%) and report lower work performance (7.0 vs. 7.4). Moreover, they are significantly more likely to be traumatized (27% vs. 17%).

**Table 2:** Descriptive statistics by conflict exposure

Variables	(1)	(2)	(3)	(4)
	Overall	Conflict	No conflict	*p*-value
Dependent variables (outcomes)				
Absenteeism (0/1)	0.248	0.285	0.225	0.010
	(0.432)	(0.019)	(0.014)	
Work performance (0–10 scale)	7.235	7.003	7.380	0.002
	(2.335)	(0.098)	(0.076)	
Mediating variable				
Trauma (0/1)	0.205	0.269	0.166	0.000
	(0.404)	(0.018)	(0.012)	
Control variables				
Male (0/1)	0.391	0.386	0.394	0.733
	(0.488)	(0.020)	(0.016)	
Age (in years)	36.427	35.759	36.847	0.064
	(11.108)	(0.450)	(0.370)	
Work experience (in years)	10.501	10.120	10.741	0.210
	(9.346)	(0.385)	(0.309)	
Married (0/1)	0.721	0.718	0.724	0.801
	(0.448)	(0.019)	(0.015)	
Primary education (0/1)	0.279	0.229	0.311	0.001
	(0.449)	(0.017)	(0.015)	
Employer is a relative (0/1)	0.184	0.189	0.181	0.683
	(0.388)	(0.0.16)	(0.013)	
Employer is a friend (0/1)	0.523	0.537	0.515	0.398
	(0.499)	(0.021)	(0.016)	
Employer is a stranger (0/1)	0.157	0.134	0.171	0.055
	(0.364)	(0.014)	(0.012)	
Respondent lives in rural areas (0/1)	0.934	0.929	0.937	0.556
	(0.248)	(0.011)	(0.008)	
Observations (N)	1,504	581	923	

*Note*: Column 1 reports summary statistics for the full sample, with standard deviation in parentheses. Columns 2 and 3 compare workers exposed to conflict with those who are not, with the corresponding p-values reported in Column 4. Key variables are measured as follows (see Methods for details). Conflict severity is measured using the Conflict Severity Index (CSI) [61, 62], derived from deadliness, danger, diffusion, and fragmentation. A dummy variable indicates with conflict (CSI = 1–4) or without conflict (CSI = 0). Trauma is assessed using the Trauma Screening Questionnaire (TSQ) [65], a validated tool for PTSD risk screening. The TSQ consists of 10 yes/no items evaluating reactions to past trauma, including intrusive thoughts, nightmares, flashbacks, hypervigilance, and emotional distress. TSQ yields a dummy variable coded as 1 if respondents are affected by trauma. We assess labor productivity through absenteeism and work performance, common proxies for health-related productivity loss [73–75]. Absenteeism is a dummy variable, coded as one if a worker logged fewer hours than committed in the past week. Work performance is measured by self-rated work performance on a 0–10 scale, with 0 as the worst and 10 as the top performance [32, 76, 77]

Additional descriptive statistics can be found in the Supplementary Material 2: Table S2 compares workers with and without trauma, while Table S3 presents the same comparison for the subsample of workers exposed to conflict. These comparisons show that traumatized workers are absent more often and report lower work performance, indicating that they have lower labor productivity than non-traumatized workers. Differences in control variables between the two groups are generally small. Table S3 further shows that, within the conflict-exposed subsample, workers with and without trauma are largely similar across most control variables.

**Fig. 3:**
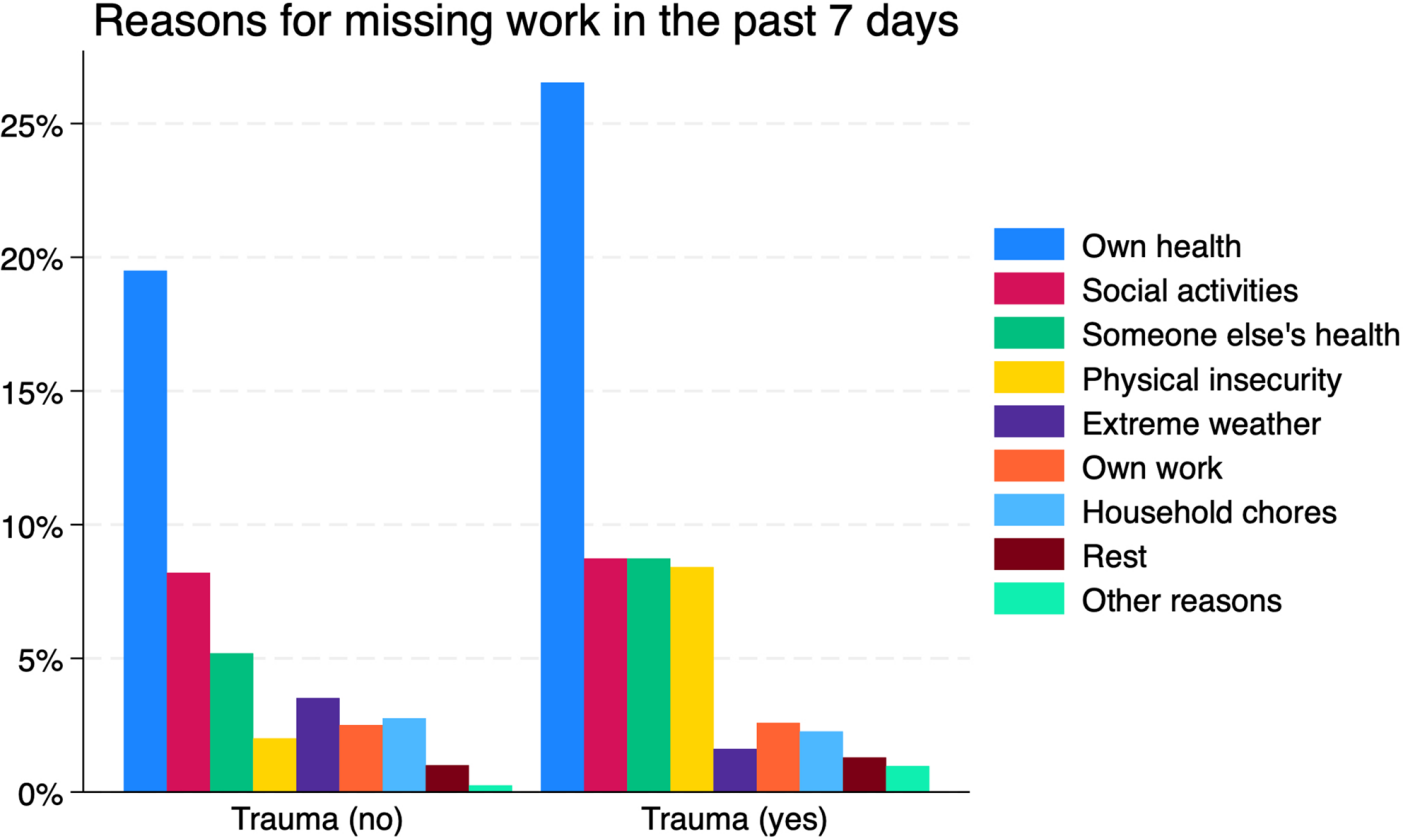
*Note*: Reasons for missing one or more days in the past 7 days, disaggregated by trauma status. The Y-axis indicates the percentage of workers who indicate one or more reasons for being absent from work at some point in the previous seven days. Mental health (trauma) is a binary indicator equal to 1 if the trauma score is ≥ 5, and 0 otherwise

Lastly, we look at common reasons for absenteeism, comparing workers with trauma and those without trauma, as shown in Fig. 3. For both groups, the primary reasons for missing work are personal health issues, followed by participation in religious or social activities, and attending to another person’s health needs. Traumatized workers additionally report concerns about physical safety during the commute to work as a key reason for absence. Overall, the reasons for absenteeism are diverse, and frequently relate to health, especially among workers affected by trauma (see Fig. 3).

### Mediation analysis results

We start by estimating the relationship between conflict and the three key variables of interest separately, with results shown in Table  3 for absenteeism (Column 1), work performance (Column 2), and mental health (Column 3). Table 3 thus presents direct associations, whereas mediation analysis [78] allows to estimate the direct, indirect, and total associations.

**Table 3:** Association between conflict and absenteeism (left), work performance (middle), and mental health (right)

		(1)		(2)		(3)
	Absenteeism		Work performance		Mental health	
	Beta	p-value	Beta	P-value	Beta	p-value
Conflict Severity Index (0/1)	0.078	0.002	-0.205	0.151	0.093	0.001
	(0.026)		(0.143)		(0.028)	
Male (0/1)	-0.002	0.936	0.147	0.262	-0.034	0.085
	(0.025)		(0.131)		(0.020)	
Age (in years)	-0.001	0.315	-0.013	0.065	-0.000	0.927
	(0.001)		(0.007)		(0.001)	
Work experience (in years)	0.001	0.631	0.024	0.002	0.001	0.324
	(0.001)		(0.008)		(0.001)	
Married (0/1)	0.017	0.496	0.296	0.033	0.014	0.582
	(0.025)		(0.138)		(0.025)	
Primary education (0/1)	-0.025	0.305	0.226	0.048	-0.015	0.560
	(0.025)		(0.114)		(0.026)	
Employer is a relative (0/1)	0.040	0.319	0.104	0.680	0.040	0.336
	(0.040)		(0.251)		(0.041)	
Employer is a friend (0/1)	0.003	0.933	0.507	0.014	0.027	0.431
	(0.033)		(0.206)		(0.034)	
Employer is a stranger (0/1)	0.077	0.086	0.482	0.037	0.117	0.019
	(0.045)		(0.230)		(0.050)	
Respondent lives in rural areas (0/1)	-0.043	0.363	0.427	0.052	0.020	0.609
	(0.048)		(0.219)		(0.039)	
*Location fixed effects*	*Yes*		*Yes*		*Yes*	
Observations (N)	1,484		1,504		1504	

*Note*: Beta = coefficient. Standard errors in parentheses, clustered at the township level. Location fixed effects are included at the second administrative level (7 states, 7 regions, and 1 union territory). Conflict is measured as a binary variable, taking the value 1 if the Conflict Severity Index (CSI) is greater than 0 and 0 if CSI equals 0. Mental health is a binary indicator equal to 1 if the trauma score is ≥ 5, and 0 otherwise. Absenteeism is also measured as a binary indicator equal to 1 if any hours were missed in the past 7 days, and 0 otherwise. Work performance is measured as a continuous variable (0–10). Mental health is a binary indicator equal to 1 if the trauma score is ≥ 5, and 0 otherwise

Mediation results are reported in Table 4, with absenteeism shown on the left and work performance on the right. A complementary, graphical illustration of the main results is provided in Fig. S1 in the Supplementary Material 2.

Starting with the direct associations, we find that conflict exposure has a direct positive association with absenteeism and a direct negative association with work performance. Conflict-exposed workers are around 7% points more likely to be absent from work and report work performance scores that are 0.15 points lower than those of non-exposed workers.

Table 4 also shows a significant indirect association between conflict and productivity, indicating that mental health—specifically trauma—amplifies the conflict–productivity relationship. Because of the significant indirect association, the total association between conflict and absenteeism is about 18% larger than when trauma is not considered as a mediator. However, conflict exposure and work performance are not statistically significant in our main specification, which includes location fixed effects. To explore this further, we compare results with location fixed effects (Table 4 below) and without fixed effects (Table S4, Columns 1 vs. 3 in the Supplementary Material 2). Estimates for absenteeism are stable in magnitude and statistical significance (left panel of Table S4 in the Supplementary Material 2), while estimates for work performance are slightly attenuated (right panel Column 1 vs. 3). Thus, there is minor variation in coefficient magnitudes, but patterns are broadly the same, irrespective of the inclusion of location fixed effects.

**Table 4:** Direct and indirect associations between conflict, mental health, and absenteeism (left) and work performance (right)

	Absenteeism		Work performance	
	Beta	*p*-value	Beta	*p*-value
Direct association	0.066	0.017	-0.152	0.292
	(0.028)		(0.144)	
Indirect association	0.012	0.046	-0.042	0.093
	(0.006)		(0.025)	
Total association	0.078	0.003	-0.194	0.170
	(0.027)		(0.141)	
*Controls*	*Yes*		*Yes*	
*Location fixed effects*	*Yes*		*Yes*	
Observations (N)	1,484		1,504	

*Note*: Beta = coefficient. Standard errors in parentheses, clustered at the township level. Location fixed effects are included at the second administrative level (7 states, 7 regions, and 1 union territory). Conflict is measured as a binary variable, taking the value 1 if the Conflict Severity Index (CSI) is greater than 0 and 0 if CSI equals 0. Mental health is a binary indicator equal to 1 if the trauma score is ≥ 5, and 0 otherwise. Absenteeism is also measured as a binary indicator equal to 1 if any hours were missed in the past 7 days, and 0 otherwise. Work performance is measured as a continuous variable (0–10)

### Sensitivity analyses

A series of robustness checks shows that our results are largely robust to alternative specifications, variables, and tests. First, we repeat the main mediation analysis (Table 4) and show in the Supplementary Material 2: treat the CSI as a continuous variable (Table S5) or use conflict-related fatalities rather than the CSI as an alternative proxy for conflict exposure (Table S6). Results largely mirror results from the main specification, which show clearer relationships for absenteeism than for work performance.

Second, we extend the conflict timeframe from three months to six months and twelve months. The results, presented in the Supplementary Material 2 (Table S7 and Fig. S2 for the six-month specification; Table S8 and Fig. S3 for the twelve-month specification), largely indicating robustness to the temporal definition of conflict exposure.

Third, we measure conflict exposure using a CSI within a 50 km radius rather than township-level conflict status (Table S9 in the Supplementary Material 2). We selected a 50 km radius, as the distances between townships in Myanmar are relatively large, and using a smaller radius would yield an insufficient number of observations. In these specifications, we clustered standard errors at a 50 km buffer level. While the results for work performance remain consistent with the main model, the model examining absenteeism is less precise. Neither the direct nor indirect association between conflict and absenteeism is statistically significant.

Fourth, we treat absenteeism as a continuous variable and show in the Supplementary Material 2: performance as a binary outcome (Table S10), and mental health as a continuous variable (Table S11), also not altering the conclusions from our main analysis. Relatedly, we use an alternative mental health measure—mental distress captured via the Hopkins Symptom Checklist (HSCL-10), which assesses anxiety and depression (Table S12). Under this specification, the indirect association is no longer statistically significant, suggesting that the relationship between conflict and work productivity is mediated through trauma, but not through anxiety and depression.

Finally, we rerun the mediation analysis for the subpopulation of workers who missed one or more workdays due to health reasons (Fig. 2), excluding those absent for other reasons (Table S13 in the Supplementary Material 2). The results are again consistent with the main analysis.

### Gendered analysis

Descriptive statistics comparing women and men are displayed in the Supplementary Material 2 (Table S14) and show that women are more likely to be exposed to conflict (i.e. 62% vs. 38% of men in conflict-exposed workers, *N* = 581), more likely to report trauma (36% vs. 29% among men). Women also display slightly higher absenteeism and lower work performance, although these differences are not statistically significant. Given the limited sample size for formal subgroup analysis, we do not pursue further econometric heterogeneity tests. More generally, our data limitations make it difficult to robustly assess gendered differences in the impacts of conflict on mental health and labor outcomes. Future research using larger samples and designs better suited for subgroup identification would be needed to more rigorously examine gendered dimensions of conflict exposure, mental health, and labor productivity.

## Discussion

The rise in violent conflict around the world hampers progress towards the SDGs, including productive employment and mental health, the lack of which further exacerbates development challenges and post-war recovery. A growing literature focuses on the implications of conflict for poverty, farming, and food security [84]. Yet socio-physiological outcomes such as mental health and labor productivity have received insufficient attention [25]. We contribute to this emerging body of literature by examining the role of mental health and labor productivity, focusing on farm labor as a key driver of food production and livelihoods.

Our descriptive statistics highlight the high prevalence of mental health issues among hired farm workers in Myanmar, as 20% experience trauma, highlighting the healthcare needs of these essential workers. This aligns with the recent assessments by the WHO regarding the prevalence of mental disorders, including depression, anxiety, and PTSD in conflict settings. Specifically, WHO estimates that approximately 22% of conflict-affected populations experience such disorders at any point in time [6]. Similarly, the prevalence rate of trauma and mental distress in our study mirrors that of the traumatic events (16% or more) among Myanmar refugees in Malaysia [85].

Our results underscore the link between conflict and mental health, in line with the existing evidence [6, 14, 85]. Conflict-affected populations often grapple with multiple challenges, including unemployment, poverty, and food insecurity, as well as direct danger from fights and limited access to basic services such as clean water, healthcare, and education [86, 87] . These traumatic experiences can profoundly influence the psychological well-being of conflict-affected populations, sometimes lasting a lifetime [86, 88, 89].

Furthermore, our results highlight that poor mental health can be a pathway through which conflict affects labor productivity, adding a novel labor focus to the literature on how conflict undermines food security. Our results are also in line with the literature on mental health and labor productivity in high-income countries [25, 26, 28]. Individuals grappling with mental health issues often encounter challenges across various aspects of their daily lives, including in their households, communities, and occupational spheres [90]. The pervasive stigma surrounding mental illness can exacerbate mental health, further impacting interpersonal and professional relationships [91]. The literature shows that armed conflict has gendered impacts, though patterns vary across contexts and outcomes [92]. Studies highlight both greater vulnerability among women to adverse social and economic effects, and differentiated labor market adjustments between men and women, including changes in hours worked and sectoral allocation [42, 92]. Overall, evidence suggests that conflict reshapes economic activity and wellbeing in gender-specific ways, rather than affecting men and women uniformly.

Our study has limitations that point to potential avenues for future research. While phone surveys were the only feasible option to obtain data in conflict-affected Myanmar, we acknowledge potential limitations. For example, phone-collected data can be subject to higher non-response and higher attrition rates compared with data collected through in-person surveys [93, 94]. This survey design (sampling strategy and phone survey) has several implications for the statistical representativeness of our data. Most importantly, our sample targets hired farm workers in Myanmar, reachable by phone. Consequently, our data does not represent individuals who have migrated abroad, were killed, or were not reachable via phone for other reasons, e.g., the destruction of communication infrastructure. This raises the possibility of survival bias, whereby the most severely affected or most vulnerable individuals may be underrepresented in our sample. If such selective non-coverage occurred, our estimates may be conservative, potentially understating the true associations between conflict, mental health and labor productivity. Thus, our approach provides nationwide data from a traditionally overlooked demographic. The limitations reflect common problems in surveys, especially those conducted in conflict-affected areas and/or via phone. However, recent analyses highlight that carefully planned phone surveys can yield high-quality data [54, 56].

While our mediation analysis provides important insights into potential pathways, it does not permit causal inference. Establishing causal links is inherently challenging in this context due to the complex and multifaceted factors that may influence both exposure to conflict and mental health outcomes. Experimental data are not available as conflict cannot be randomized, explaining the prevalence of observational studies in this area. Panel data would be a step into mitigating this concern, though data collection in conflict settings is generally challenging, but is feasible, as we describe in the data section. Beyond data and methodological issues, future research could explore additional channels (e.g., food insecurity, physical injury, etc.) that may mediate the relationship between conflict and labor productivity.

## Conclusions

Our study reinforces the case for mental health interventions in conflict-affected settings, including in remote, agricultural communities. This could include: (1) incorporating psychosocial support components in agricultural extension services; (2) adopting policy frameworks that link conflict mitigation, mental health services, and agricultural productivity enhancement; (3) implementing practical approaches to reach vulnerable workers with limited access to healthcare infrastructure; and (4) designing gender-sensitive mental health strategies [95, 96], recognizing that gender norms can influence healthcare access to and utilization of healthcare services.

As evidence of the negative implications of conflict is mounting, more research is needed on how to target and support vulnerable populations to sustain mental health, livelihoods, and food production.

## Supplementary Information

Below is the link to the electronic supplementary material.

Supplementary Material 1: Agricultural Laborer Survey Phone Survey in Myanmar. The full questionnaire used for the data collection in the study.

Supplementary Material 2: Supplementary Results. Includes descriptive statistics, robustness checks, and additional supporting analyses not included in the main manuscript.

Supplementary Material 3: The Stata Code for Analysis. Stata do-file used for statistical analyses.

## Data Availability

The data described in this article can be freely and openly available from the ETH Zurich Research Collection [97] at https://doi.org/10.3929/ethz-c-000798894. The survey questionnaire is provided as Supplementary Material 1, supplementary results as Supplementary Material 2, and the Stata code used for the analyses as Supplementary Material  3.

## Abbreviations

ACLED: Armed Conflict Location and Event Data
CATI: Computer-Assisted Telephone Interviews
CSI: Conflict Severity Index
HSCL-10: Hopkins Symptom Checklist-10
WHO-HPQ: World Health Organization Health and Work Performance Questions
IFPRI: International Food Policy Research Institute
MHWS: Myanmar Household Welfare Survey
PTSD: Post-Traumatic Stress Disorder
SDGs: Sustainable Development Goals
TSQ: Trauma Screening Questionnaire
