# Supplementary material for: Conflict, mental health, and labor productivity: evidence from hired farm workers in Myanmar
Source: BMC Glob Public Health. 2026 Jul 29;4:72. doi: 10.1186/s44263-026-00307-5 (PMC13421825; doi:10.1186/s44263-026-00307-5)
Supplement: Supplementary file 1 — Supplementary Material 1: Agricultural Laborer Survey Phone Survey in Myanmar. The full questionnaire used for the data collection in the study. [file 44263_2026_307_MOESM1_ESM.docx]

**AGRICULTURAL LABORER PHONE SURVEY IN MYANMAR**

Final Questionnaire – November 26, 2023

# MODULE A: Call Information

(module can be adjusted by phone survey company if XXX agrees)

*Outcomes and time code (HH:MM) should be recorded for each attempted call. Follow-ups attempts should be made at different times of day than previous attempts. Enumerator’s name and code should be recorded for each attempt.*

| **A.01** Respondent identifier number [01-0000] | *(Number)* |
| --- | --- |
| **A.02** Enumerator name and code | *(Name and code)* |
| **A.03** 1^st^ attempt: Is the respondent able to talk? | 1 = Yes  2 = Did not answer  3 = Wrong number  4 = Declined to participate  9999 = Other (*specify)* ________________ |
| **A.04** 1^st^ attempt: Time of day | *(HH:MM)*  **If A.03 == 1 >> Module B** |
| **A.05** 2^nd^ attempt: Is the respondent able to talk? | 1 = Yes  2 = Did not answer  3 = Wrong number  4 = Declined to participate  9999 = Other (*specify)* ________________ |
| **A.06** 2^nd^ attempt: Time of day | *(HH:MM)*  **If A.05 == 1 >> Module B** |
| **A.07** 3^rd^ attempt: Is the respondent able to talk? | 1 = Yes  2 = Did not answer  3 = Wrong number  4 = Declined to participate  9999 = Other (*specify)* ________________ |
| **A.08** 3^rd^ attempt: Time of day | *(HH:MM)*  **If A.07 == 1 >> Module B** |
| **A.09** 4^th^ attempt: Is the respondent able to talk? | 1 = Yes  2 = Did not answer  3 = Wrong number  4 = Declined to participate  9999 = Other (*specify)* ________________ |
| **A.10** 4^th^ attempt: Time of day | *(HH:MM)*  **If A.09 == 1 >> Module B** |
| **A.11** 5^th^ attempt: Is the respondent able to talk? | 1 = Yes  2 = Did not answer  3 = Wrong number  4 = Declined to participate  9999 = Other (*specify)* ________________ |
| **A.12** 5^th^ attempt: Time of day | *(HH:MM)*  **If A.11 == 1 >> Module B** |

| **Introduction and informed consent form**  My name is ___________________ and I work for MSR. Last month, we contacted your household to discuss a number of livelihood and welfare questions. We are calling you now to follow up with another telephone survey interview related to your employment. We are conducting this survey for the International Food Policy Research Institute (IFPRI) and ETH Zurich. As before, your responses will be kept completely confidential and will be combined with responses from others all over the country. The information we received from all our respondents was very valuable, and it is important to keep assessing the status of employment conditions in the agricultural sector in the country as well as the impacts of the recent events in the country and inform the private sector, civil society, and international assistance agencies on ways to improve the livelihoods of Myanmar people.  In the survey, we will not inquire about your personal information such as your age, gender, education and total household member. IFPRI will be sharing with us such information collected in six different rounds of the Myanmar Household Welfare Survey (MHWS). During this call, we would like to ask you about your mental health conditions and trauma experiences, as well as your livelihood conditions in the recent farming seasons. We understand that you may or may not experience discomforts, including feelings of sadness, anxiety, or anger while answering questions related to your mental health and trauma experiences.  Your participation is voluntary. You can stop participating at any time. During the interview, you may also refuse to answer any questions at any time without fear of losing any rights to which you are entitled. The interview will take approximately 50 minutes. There is no risk in your participation, except the 50 minutes you will spend answering our questions. We do not intend to ask any controversial questions. As a token of appreciation, upon completion of the interview, we will send a 6,000-kyat phone credit to this phone number after the interview.  The analysis results, combining data from all participants, including yours, will be used in a Ph.D. thesis and scientific journal article in an aggregated and anonymized form. This ensures that your information cannot be identified by the reader of these publications. We will delete your personal data at the end of the project (04-05-2026).  If you have any questions about your rights as a research participant, please contact the ETH Zurich Ethics Commission, at [ethics@sl.ethz.ch](mailto:ethics@sl.ethz.ch) or 0041 44 632 85 72 or Olivette Burton, IFPRI IRB Coordinator, at [ifpri-irb@cgiar.org](mailto:ifpri-irb@cgiar.org). For any questions regarding this research, please contact the Myanmar Survey Office in Yangon at +95 1 8370464.  Do you agree to participant in this survey? If your answer is ‘yes,’ we continue with the survey questionnaire. If your answer is ‘no,’ we will conclude the interview. |
| --- |

# MODULE B: AGRICULTURAL WORK ACTIVITIES IN THE PAST 12 MONTHS

| **B.01** Did you work as an agricultural worker in the past 12 months?  [We believe we are calling to farm laborers who identified themselves in the previous MHWS surveys] | 1= Yes  2= No **>> STOP INTERVIEW** |
| --- | --- |
| **B.02** How many years you have been working as an agricultural laborer?  [Agricultural work means all tasks happened on the field, from tillage and ploughing land to harvesting crops until harvested crops are loaded on the truck and stored in warehouse of farmer] | 1. ___ years number  [If the respondent provides the age or year that he/she started working as a laborer, calculate the work experience using his age] |
| **B.03** What other income-generating activities do you usually do besides agricultural job? | 1 = wage work – non-agriculture  2 = salaried work– non-agriculture  3 = work on the own or household crop farm (seasonal and perennial crops)  4 = own or household livestock business  5 = own or household fishing or aquaculture business  6 = own or a household non-farm enterprise (including any small business activities)  7 = renting out of land / properties; interest from loans (if not as lending business) – from bank or private lenders …  8 = gifts, donations, pensions, assistance  9 = remittances  10 = no employment and no income |
| **B.04** Is your spouse (husband/wife) is also an agricultural laborer?  [If the respondent is unmarried] | 1= Yes  2= No  9996 = Unmarried |
| **B.05** What other income-generating activities do your spouse(husband/wife) usually do besides agricultural job? | 1 = wage work – non-agriculture  2 = salaried work– non-agriculture  3 = work on the own or household crop farm (seasonal and perennial crops)  4 = own or household livestock business  5 = own or household fishing or aquaculture business  6 = own or a household non-farm enterprise (including any small business activities)  7 = renting out of land / properties; interest from loans (if not as lending business) – from bank or private lenders …  8 = gifts, donations, pensions, assistance  9 = remittances  10 = no employment and no income |

| **RECENT RAINY SEASON [mid-May 2023 – late-Oct 2023] NAYON-WASO-WAHGAUNG-TAWTHALIN** | | | | | | | | | | | | | |
| --- | --- | --- | --- | --- | --- | --- | --- | --- | --- | --- | --- | --- | --- |
| **B.06** How difficult is it to find an agricultural job in the recent monsoon season? | 1=Very difficult  2=Somewhat difficult  3=No difficulty  4=Did not do any agricultural work in this season **>> Next season** | | | | | | | | | | | | |
| **B.07** How did you usually find agricultural jobs in the recent monsoon season?  B.07A What was the most common?  CAPI: preload options selected in B.07A, allow only one of those to be selected. | 1= Through the employer directly  2= Through family/friends/relatives/villagers  3= Through regularly working group member  4= Through co-workers from previous non-agricultural work  5= Through employer/supervisor from previous non-agricultural work  6= Through agent/job agency  7= Other, specify: ____ | | | | | | | | | | | | |
| **B.08** How many different farms (employers) you have been working for in the recent monsoon season? | 1= _____number | | | | | | | | | | | | |
| **FARM 1** | | | | | | | | | | | | | |
| **B.09** Please tell us the details of farm 1 you worked for in the recent monsoon season.  [If respondent worked for more than 1 farmer in one season, ask the 1 farm that the respondent worked for longest days] | What task? | Days worked? | | Money received? | | Payment rate? | | Size of land areas owned by farmer? | | Relation with owner? | | Gender of owner/major decision maker? | Township name |
|  | (#code) |  | |  | | (#code) | |  | |  | |  | Pre-load |
|  |  | __days | | __mmk | |  | | 1. Acre  2. length x width (feet)  3. length x width (taung) | | 1=Relatives  2=Friends/neighbor/villager  3=Other/stranger | | 1=Male  2=Female | < preload list of S/R; Shan State by North, East and South >  + add another S/R (specify 1,2,3) |
| **B.10** Do you get paid in kind (e.g., crops and any other items) for work done? | 1= Yes  2= No **>> B.11** | | What crop/item? | | Amount/volume? | | | | Weight unit? | | Amount in kyat per {Weight unit} | | |
|  |  |  | (#code)  Capi: loop up to 3 most important crops | |  | | | | (#code) | | ________ | | |
|  |  |  |  | | _______ | | | |  | |  | | |
| **B.11** Did you work some days during this period for farm 1 to **pay off debt?** | 1= Yes from farmer 12= No **>> B.16**  3= Yes from group leaders (don’t know which farms) _ | | Type of debt/loan you took? | | Kyat/crop amount? | | | | Total days worked in pre-agreed rate? | | Is pre-agreed rate being lower/higher than market rate? | | If lower/higher, how much? |
|  |  |  |  | | (#code) | | Crop name & unit? | |  | |  | |  |
|  |  |  | 1= Cash  2= Crop  3= Both | | 1=_____mmk  2=___in kind | | 1.___name  2. ___unit | | 1=__days  2=__Acre  3= No agreement, paid by market rate **>> B.16** | | 1=Lower  2=Same **>> B.12**  3=Higher | | 1=___mmk/per day  2=___mmk/per acre |
| **B.12** Has the initially planned agreements including task, days, and working hours been mostly honored or not by farm owner? | 1= Yes  2= No  3= I do not know | | | | | | | | | | | | |
| **B.13** Did you leave before the agreed-upon duration was fulfilled? | 1= Yes  2= No **>> B.15**  96 = Do not want to tell **>> B.16** | | | | | | | | | | | | |
| **B.14** If yes, why?  [multi-select] | 1= Debt repaid (labor days or task)  2= Found a better-paid job  3= Employer dishonors the initial agreement wages/working conditions  4= Had to flee due to insecurity  5= Bad relationship with farmer  6= Others, specify: ___ | | | | | | | | | | | | |
| **B.15** Why not?  [multi-select] | 1= Until debt repaid  2= Good wages  3= Agreed wages/working conditions are honored by owner  4= Others, specify: ___ | | | | | | | | | | | | |
| **B.16** Do you have any plans to return to work for this employer  [farm 1]? | 1= Yes  2= No **>> B.18**  96 = Do not want to tell **>> B.19** | | | | | | | | | | | | |
| **B.17** If yes, why?  [multi-select] | 1= Haven’t found a better paid job  2= Acceptable working conditions  3= Good relationship with the farmer  4= Reduced violence/insecurity  5= Others, specify: ___ | | | | | | | | | | | | |
| **B.18** Why not?  [multi-select] | 1= Low wages  2= Work finished  3= Bad working conditions/bad relationship with farmer  4= Others, specify: ___ | | | | | | | | | | | | |
| **B.19** How many hours do you usually work per day? | 1= ____hours__min  2= ____am to ____p.m.  3= Do not know | | | | | | | | | | | | |
| **B.20** How many hours of break time do you usually have in a working day (including lunch time)? | 1= ____hours__min  2= ____a.m____p.m  3= Do not know | | | | | | | | | | | | |
| **B.21** In the case you work with chemicals or assign to risky tasks, do you work usually with protected equipment? | 1= Yes (Provided)  2= Yes (No provided)  3= No (No provided)  4= No (Provided)  5= Not applicable | | | | | | | | | | | | |
| **B.22** Do you usually receive job-related, including safety training? | 1= Yes  2= No (skip B23) | | | | | | | | | | | | |
| **B.23** Number of hours/days you receive job-related training? | 1=___hours  2=___days | | | | | | | | | | | | |

| **B.24** How many days did you work more for other farmers in total in this MONSOON season? | Average or common amount of wage? | Payment rate? |
| --- | --- | --- |
| __days | __mmk | (#code) |

| **SUMMER SEASON [ mid-Feb 2023 – mid-May 2023] TABAUNG-TAGU-KASON** | | | | | | | | | | | | | | |
| --- | --- | --- | --- | --- | --- | --- | --- | --- | --- | --- | --- | --- | --- | --- |
| **B.06** How difficult is it to find an agricultural job in this season? | 1=Very difficult  2=Somewhat difficult  3=No difficulty  4=Did not do any agricultural work in this season **>> Next season** | | | | | | | | | | | | | |
| **B.07** How did you usually find agricultural jobs in this season?  B.07A What was the most common?  CAPI: preload options selected in B.07A, allow only one of those to be selected. | 1= Through the employer directly  2= Through family/friends/relatives/villagers  3= Through regularly working group member  4= Through co-workers from previous non-agricultural work  5= Through employer/supervisor from previous non-agricultural work  6= Through agent/job agency  7= Other, specify: ____ | | | | | | | | | | | | | |
| **B.08** How many different farms (employers) you have been working for in this season? | 1= _____number | | | | | | | | | | | | | |
| **B.08** {$B.08A} How many farms are the same farm from the rainy season?  **(If Bo6=code 4 in rainy season, skip this question)** | 1= _____number | | | | | | | | | | | | | |
| **FARM 1** | | | | | | | | | | | | | | |
| **B.09** Please tell us the details of farm 1 you worked for in this season.  [If respondent worked for more than 1 farmer in one season, ask the 1 farm that the respondent worked for longest days] | What task? | Days worked? | | Money received? | Payment rate? | | | | Size of land areas owned by farmer? | | Relation with owner? | | Gender of owner/major decision maker? | Township name |
|  | (#code) |  | |  | (#code) | | | |  | |  | |  | Pre-load |
|  |  | __days | | __mmk |  | | | | 1. Acre  2. length x width (feet)  3. length x width (taung) | | 1=Relatives  2=Friends/neighbor/villager  3=Other/stranger | | 1=Male  2=Female  3=Both | < preload list of S/R; Shan State by North, East and South >+ add another S/R (specify 1,2,3) |
| **B.10** Do you get paid in kind (e.g., crops and any other items) for work done? | 1= Yes  2= No **>> B.11** | | What crop/item? | | | | Amount/volume? | | | Weight unit? | | Amount in kyat per {Weight unit} | | |
|  |  |  | (#code)  Capi: loop up to 3 most important crops | | |  | | | | (#code) | | ________ | | |
|  |  |  |  | | | _______ | | | |  | |  | | |
| **B.11** Did you work some days during this period for farm 1 to **pay off debt?** | 1= Yes  2= No **>> B.16**  3= Yes from group leaders (don’t know which farms) _ | | Type of debt/loan you took? | | | Kyat/crop amount? | | | | Total days worked in pre-agreed rate? | | Is pre-agreed rate being lower/higher than market rate? | | If lower/higher, how much? |
|  |  |  |  | | | (#code) | | Crop name & unit? | |  | |  | |  |
|  |  |  | 1= Cash  2= Crop  3= Both | | | 1=_____mmk  2=___in kind | | 1.___name  2. ___unit | | 1=__days  2=__Acre  3= No agreement, paid by market rate **>> B.16** | | 1=Lower  2=Same **>> B.12**  3=Higher | | ___mmk |
| **B.12** Has the initially planned agreements including task, days, and working hours been mostly honored or not? | 1= Yes  3= No  2= I do not know | | | | | | | | | | | | | |
| **B.13** Did you leave before the agreed-upon duration was fulfilled? | 1= Yes  2= No **>> B.15**  96 = Do not want to tell **>> B.16** | | | | | | | | | | | | | |
| **B.14** If yes, why?  [multi-select] | 1= Debt repaid (labor days or task)  2= Found a better-paid job  3= Employer dishonors the initial agreement wages/working conditions  4= Had to flee due to insecurity  5= Bad relationship with farmer  6= Others, specify: ___ | | | | | | | | | | | | | |
| **B.15** Why not?  [multi-select] | 1= Until debt repaid  2= Good wages  3= Agreed wages/working conditions are met  4= Others, specify: ___ | | | | | | | | | | | | | |
| **B.19** How many hours do you usually work per day? | 1= ____hours__min  2= ____a.m____p.m  3= Do not know | | | | | | | | | | | | | |
| **B.20** How many hours of break time do you usually have in a working day (including lunch time)? | 1= ____hours__min  2= ____a.m____p.m  3= Do not know | | | | | | | | | | | | | |
| **B.21** In the case you work with chemicals or assign to risky tasks, do you work usually with protected equipment? | 1= Yes (Provided)  2= Yes (No provided)  3= No (No provided)  4= No (Provided)  5= Not applicable | | | | | | | | | | | | | |
| **B.22** Do you usually receive job-related, including safety training? | 1= Yes  2= No | | | | | | | | | | | | | |
| **B.23** Number of hours/days you receive job-related training? | 1=___hours  2=___days | | | | | | | | | | | | | |

| **B.24** How many days did you work more for other farmers in total in this SUMMER season? | Average or common amount of wage? | Payment rate? |
| --- | --- | --- |
| __days | __mmk | (#code) |

| **WINTER SEASON [late-Oct 2022 – mid-Feb 2023] TAZAUNGMON-NATDAW-PYATHOE-TABODWE** | | | | | | | | | | | | | |
| --- | --- | --- | --- | --- | --- | --- | --- | --- | --- | --- | --- | --- | --- |
| **B.06** How difficult is it to find an agricultural job in this season? | 1=Very difficult  2=Somewhat difficult  3=No difficulty  4=Did not do any agricultural work in this season **>> Next season** | | | | | | | | | | | | |
| **B.07** How did you usually find agricultural jobs in this season?  B.07A What was the most common?  CAPI: preload options selected in B.07A, allow only one of those to be selected. | 1= Through the employer directly  2= Through family/friends/relatives/villagers  3= Through regularly working group member  4= Through co-workers from previous non-agricultural work  5= Through employer/supervisor from previous non-agricultural work  6= Through agent/job agency  7= Other, specify: ____ | | | | | | | | | | | | |
| **B.08** How many different farms (employers) you have been working for in this season? | 1= _____number | | | | | | | | | | | | |
| **B.08** {$B.08A} How many of these firms are the same firms in the summer and rainy (current) season? | 1= _____number | | | | | | | | | | | | |
| **FARM 1** | | | | | | | | | | | | | |
| **B.09** Please tell us the details of farm 1 you worked for in this season.  [If respondent worked for more than 1 farmer in one season, ask the 1 farm that the respondent worked for longest days] | What task? | Days worked? | | Money received? | | Payment rate? | | Size of land areas owned by farmer? | | Relation with owner? | | Gender of owner/major decision maker? | Township name |
|  | (#code) |  | |  | | (#code) | |  | |  | |  | Pre-load |
|  |  | __days | | __mmk | |  | | 1. Acre  2. length x width (feet)  3. length x width (taung) | | 1=Relatives  2=Friends/neighbor/villager  3=Other/stranger | | 1=Male  2=Female  3=Both | < preload list of S/R; Shan State by North, East and South > + add another S/R (specify 1,2,3) |
| **B.10** Do you get paid in kind (e.g., crops and any other items) for work done? | 1= Yes  2= No **>> B.11** | | What crop/item? | | Amount/volume? | | | | Weight unit? | | Amount in kyat per {Weight unit} | | |
|  |  |  | (#code)  Capi: loop up to 3 most important crops | |  | | | | (#code) | | ________ | | |
|  |  |  |  | | _______ | | | |  | |  | | |
| **B.11** Did you work some days during this period for farm 1 to **pay off debt?** | 1= Yes  2= No **>> B.16**  3= Yes from group leaders (don’t know which farms) | | Type of debt/loan you took? | | Kyat/crop amount? | | | | Total days worked in pre-agreed rate? | | Is pre-agreed rate being lower/higher than market rate? | | If lower/higher, how much? |
|  |  |  |  | | (#code) | | Crop name & unit? | |  | |  | |  |
|  |  |  | 1= Cash  2= Crop  3= Both | | 1=_____mmk  2=___in kind | | 1.___name  2. ___unit | | 1=__days  2=__Acre  3= No agreement, paid by market rate **>> B.16** | | 1=Lower  2=Same **>> B.12**  3=Higher | | ___mmk |
| **B.12** Has the initially planned agreements including task, days, and working hours been mostly honored or not? | 1= Yes  3= No  2= I do not know | | | | | | | | | | | | |
| **B.13** Did you leave before the agreed-upon duration was fulfilled? | 1= Yes  2= No **>> B.15**  96 = Do not want to tell **>> B.16** | | | | | | | | | | | | |
| **B.14** If yes, why?  [multi-select] | 1= Debt repaid (labor days or task)  2= Found a better-paid job  3= Employer dishonors the initial agreement wages/working conditions  4= Had to flee due to insecurity  5= Bad relationship with farmer  6= Others, specify: ___ | | | | | | | | | | | | |
| **B.15** Why not?  [multi-select] | 1= Until debt repaid  2= Good wages  3= Agreed wages/working conditions are met  4= Others, specify: ___ | | | | | | | | | | | | |
| **B.19** How many hours do you usually work per day? | 1= ____hours__min  2= ____a.m____p.m  3= Do not know | | | | | | | | | | | | |
| **B.20** How many hours of break time do you usually have in a working day (including lunch time)? | 1= ____hours__min  2= ____a.m____p.m  3= Do not know | | | | | | | | | | | | |
| **B.21** In the case you work with chemicals or assign to risky tasks, do you work usually with protected equipment? | 1= Yes (Provided)  2= Yes (No provided)  3= No (No provided)  4= No (Provided)  5= Not applicable | | | | | | | | | | | | |
| **B.22** Do you usually receive job-related, including safety training? | 1= Yes  2= No | | | | | | | | | | | | |
| **B.23** Number of hours/days you receive job-related training? | 1=___hours  2=___days | | | | | | | | | | | | |

| **B.24** How many days did you work more for other farmers in total in this WINTER season? | Average or common amount of wage? | Payment rate? |
| --- | --- | --- |
| __days | __mmk | (#code) |

| **APPLIES TO ALL FARMERS THAT YOU WORKED FOR IN EACH SEASON** | | | | | | | | | | | | | | | | | | | | | | |
| --- | --- | --- | --- | --- | --- | --- | --- | --- | --- | --- | --- | --- | --- | --- | --- | --- | --- | --- | --- | --- | --- | --- |
|  | Rainy season  [mid-May – late-Oct] Now | | | | | | | | Summer  [ mid-Feb – mid-May] | | | | | | | | Winter  [late-Oct – mid-Feb] | | | | | |
| **#** | # | | | | | | | | # | | | | | | | | # | | | | | |
| **B.24** How is the pay rate typically determined for the tasks you performed regardless of the payment method? | 1= Negotiated before work  2= Fixed community pay rate  3= Others, specify: ___ | | | | | | | | 1= Negotiated before work  2= Fixed community pay rate  3= Others, specify: ___ | | | | | | | | 1= Negotiated before work  2= Fixed community pay rate  3= Others, specify: ___ | | | | | |
| **B.25** How much negotiation power do you have over your pay rate? | 1= No negotiable **>> B.28**  2= Very low  3= Neutral  4= Very high | | | | | | | | 1= No negotiable **>> B.28**  2= Very low  3= Neutral  4= Very high | | | | | | | | 1= No negotiable **>> B.28**  2= Very low  3= Neutral  4= Very high | | | | | |
| **B.26** Who negotiated the wages and pay rate?  [Group means someone or a senior invite you to work on a farm for one or more days] | 1= By myself  2= Group leader/member  3= Others, specify: ___ | | | | | | | | 1= By myself  2= Group leader/member  3= Others, specify: ___ | | | | | | | | 1= By myself  2= Group leader/member  3= Others, specify: ___ | | | | | |
| **B.27** Can you tell us by how much the wage and pay rate are increased after negotiation?  B.27A  1= Cash  2=In-kind >B.30  3= Both | Amount | | Payment rate | | | | | | Amount | | | | Payment rate | | | | Amount | | | Payment rate | | |
|  |  | | (#code) | | | | | |  | | | | (#code) | | | |  | | | (#code) | | |
|  | ____mmk | |  | | | | | | ____mmk | | | |  | | | | ____mmk | | |  | | |
| **B.28** Do you receive a cash bonus besides your wage? | 1= Yes  2= No **>> B.30** | | | | | | | | 1= Yes  2= No **>> B.30** | | | | | | | | 1= Yes  2= No **>> B.30** | | | | | |
| **B.29** The bonus amount?  [Paid in cash] | Amount | | | Payment rate | | | | | Amount | | | | | Payment rate | | | Amount | | | Payment rate | | |
|  |  | | | (#code) | | | | |  | | | | | (#code) | | |  | | | (#code) | | |
|  | ____mmk | | |  | | | | | ____mmk | | | | |  | | | ____mmk | | |  | | |
| **B.30** If in kind payment, crop/item volume increase after negotiation?  No in-kind payment **>> B.31(B.27A !=1\|, skip to B.31)** | Crop Name | Volume | | Weight unit | | Payment rate | | | Crop Name | Volume | | Weight unit | | | | Payment rate | Crop Name | Volume | | Weight unit | | Payment rate |
|  | (#code) |  | | (#code) | | (#code) | | | (#code) |  | | (#code) | | | | (#code) | (#code) |  | | (#code) | | (#code) |
|  |  | ____ | |  | |  | | |  | ____ | |  | | | |  |  | ____ | |  | |  |
| **B.31** Do you receive in kind payment (crop/item) bonus?  **B.27A !=1\|, skip to B.31)** | 1= Yes  2= No **>> B.32** | | | | | | | | 1= Yes  2= No **>> B.32** | | | | | | | | 1= Yes  2= No **>> B.32** | | | | | |
| **B.32** The bonus amount?  [In-kind payment]  **B.27A !=1\|, skip to B.31)** | Crop Name | Volume | | Weight unit | | | Payment rate | | Crop Name | | Volume | Weight unit | | | | Payment rate | Crop Name | Volume | | | Weight unit | Payment rate |
|  | (#code) |  | | (#code) | | | (#code) | | (#code) | |  | (#code) | | | | (#code) | (#code) |  | | | (#code) | (#code) |
|  |  | ____ | |  | | |  | |  | | ____ |  | | | |  |  | ____ | | |  |  |
| **B.33** How do you typically determine the daily working hour (including, break time)? | 1= Negotiated before work  2= Fixed community rate  3= No need because of task-based/piece rate  4= Others, specify: ___ | | | | | | | | 1= Negotiated before work  2= Fixed community rate  3= No need because of task-based/piece rate  4= Others, specify: ___ | | | | | | | | 1= Negotiated before work  2= Fixed community rate  3= No need because of task-based/piece rate  4= Others, specify: ___ | | | | | |
| **B.34** How much negotiation power do you have over your working hour? | 1= No negotiable  2= Very low  3= Neutral  4= Very high | | | | | | | | 1= No negotiable  2= Very low  3= Neutral  4= Very high | | | | | | | | 1= No negotiable  2= Very low  3= Neutral  4= Very high | | | | | |
| **B.35** Please tell us the non-wage benefits you receive besides your wages/salaried/crops.  [multi-select] | 1= Meals  2= No non-wage benefits **>> B.37**  3= Housing/accommodation**>> B.37**  4= Transportation**>> B.37**  5. Refreshments (tea-leaf salad, tea, coffee, soft drinks, etc.,) **>> B.37**  6= Cloths/uniform**>> B.37**  7= Cigarette/betel nut/alcohol/beer**>> B.37**  8= Health insurance/care**>> B.37**  **9 =gift for home consumption** ____**>> B.37**  10= Other, specify: ____**>> B.37** | | | | | | | | 1= Meals  2= No non-wage benefits **>> B.37**  3= Housing/accommodation**>> B.37**  4= Transportation**>> B.37**  5. Refreshments (tea-leaf salad, tea, coffee, soft drinks, etc.,) **>> B.37**  6= Cloths/uniform**>> B.37**  7= Cigarette/betel nut/alcohol/beer**>> B.37**  8= Health insurance/care**>> B.37**  **9 =gift for home consumption** ____**>> B.37**  10= Other, specify: ____**>> B.37** | | | | | | | | 1= Meals  2= No non-wage benefits **>> B.37**  3= Housing/accommodation**>> B.37**  4= Transportation**>> B.37**  5. Refreshments (tea-leaf salad, tea, coffee, soft drinks, etc.,) **>> B.37**  6= Cloths/uniform**>> B.37**  7= Cigarette/betel nut/alcohol/beer**>> B.37**  8= Health insurance/care**>> B.37**  **9 =gift for home consumption** ____**>> B.37**  10= Other, specify: ____**>> B.37** | | | | | |
| **B.36** How often is meals provided per day? | 1= One meal per day/cash for one meal  2= Two meals per day/cash for two meals  3= Three meals per day/cash three meals  4= No meal provided  5= Other, specify: ____ | | | | | | | | 1= One meal per day/cash for one meal  2= Two meals per day/cash for two meals  3= Three meals per day/cash three meals  4= No meal provided  5= Other, specify: ____ | | | | | | | | 1= One meal per day/cash for one meal  2= Two meals per day/cash for two meals  3= Three meals per day/cash three meals  4= No meal provided  5= Other, specify: ____ | | | | | |
| **B.37** Who usually supervises you at your workplace? | 1= The owner directly  2= The group leader/senior  3= The farm supervisor  4= Other, specify: ____ | | | | | | | | 1= The owner directly  2= The group leader/senior  3= The farm supervisor  4= Other, specify: ____ | | | | | | | | 1= The owner directly  2= The group leader/senior  3= The farm supervisor  4= Other, specify: ____ | | | | | |
| **B.38** Did you usually work as a group or individually or with family in each season? | 1= Individually  2= As a group  3= With family  4= Others, specify: ___ | | | | | | | | 1= Individually  2= As a group  3= With family  4= Others, specify: ___ | | | | | | | | 1= Individually  2= As a group  3= With family  4= Others, specify: ___ | | | | | |
| **B.39** The advantages of working in a group? **[capi: ask if only B.38==2)** | | | | | | | | | 1= Easy to find job  2= Higher when working on a group contract  3= Can take higher credit from employer  4= Feeling safer in group  5= Others, specify: ___ | | | | | | | | | | | | | |
| **B.40** Is the agricultural worker group common in your village tract/ward? | | | | | | | | | 1= Yes  2= No  3= I do not know | | | | | | | | | | | | | |
| **B.41** Have you participated in a worker group in the last 12 months? | | | | | | | | | 1= Yes  2= No **>> B.47** | | | | | | | | | | | | | |
| **B.42** How many different agricultural worker groups that you have participated in the respective season?  [If more than 3 groups, ask only the 3 most important groups] | _____number | | | | | | | | _____number | | | | | | | | _____number | | | | | |
| **GROUP 1** | | | | | | | | | | | | | | | | | | | | | | |
| **B.43** Please tell us the details of group 1 you have participated in? | Established year? | | | | How many members? | | | Members’ gender? | | | How long have been with the group? | | | | Your position? | | | | Group leader’s gender | | | |
|  | 1 =____month  2= ____year  3= Do not know | | | | ______ | | | 1=___male  2=___female | | | 1 =____month  2= ____year  3= Do not know | | | | 1= Leader/head of the group  2= Second head of the group  3= Just a member  4= Others, specify: ___  5= Not applicable | | | | 1=male  2=female | | | |
| **GROUP 2** | | | | | | | | | | | | | | | | | | | | | | |
| **B.44** Please tell us the details of group 2 you have participated in? | Established year? | | | | How many members? | | | Their gender? | | | How long have been with the group? | | | | Your position? | | | | Group leader’s gender | | | |
|  | 1 =____month  2= ____year  3= Do not know | | | | ______ | | | 1=___male  2=___female | | | 1 =____month  2= ____year  3= Do not know | | | | 1= Leader/head of the group  2= Second head of the group  3= Just a member  4= Others, specify: ___  5= Not applicable | | | | 1=male  2=female | | | |
| **GROUP 3** | | | | | | | | | | | | | | | | | | | | | | |
| **B.45** Please tell us the details of group 3 you have participated in? | Established year? | | | | How many members? | | | Their gender? | | | How long have been with the group? | | | | Your position? | | | | Group leader’s gender | | | |
|  | 1 =____month  2= ____year  3= Do not know | | | | ______ | | | 1=___male  2=___female | | | 1 =____month  2= ____year  3= Do not know | | | | 1= Leader/head of the group  2= Second head of the group  3= Just a member  4= Others, specify: ___  5= Not applicable | | | | 1=male  2=female | | | |

|  | Rainy season  [mid-May – late-Oct] Now | Summer  [ mid-Feb – mid-May] | Winter  [late-Oct – mid-Feb] |
| --- | --- | --- | --- |
| **B.46** Can you tell us which groups you participated in each season? | 1= Group 1  2= Group 2  3= Group 3  4= All groups  5= Group 1&2  6= Group 1&3  7= Group 2&3 | 1= Group 1  2= Group 2  3= Group 3  4= All groups  5= Group 1&2  6= Group 1&3  7= Group 2&3 | 1= Group 1  2= Group 2  3= Group 3  4= All groups  5= Group 1&2  6= Group 1&3  7= Group 2&3 |

| **B.47** What are the advantages of joining a worker group?  [multi-select]  [Enumerator: ask all respondents regardless of whether they work in a group or not] [skip if B.39==2] | 1= Easy to find work through group/employer prefer group worker  2= Wages are higher  3= Ability to take higher credits from employer  4= I feel safer in a group  5= Others, specify: ___ |
| --- | --- |
| **B.48** What are the disadvantages of joining a worker group?  [multi-select]  [Enumerator: ask all respondents regardless of whether they work in a group or not] | 1= Require flexibility to travel distance location  2= I lose freedom  3= Wages can be lower  4= Paid discrimination among members  5= Others, specify: ___ |
| **B.49** Can you tell us why some workers choose not to join a worker group (including yourself)?  [multi-select] | 1= Cannot travel to distance location  2= They prefer working individually  3= They can find work directly through farmers and others  4= They have no link to any group/there is no such worker group in their place  5= Other, specify: ___ |
| **B.50** Have you ever heard about the Agriculture and Farmers Federation of Myanmar (AFFM)? | 1= Yes  2= No |
| **B.51** Have you even been a member of the Agriculture and Farmers Federation of Myanmar (AFFM)?  [single-select] | 1= Yes  2= No  96 = Do not want to tell |
| **B.52** What are the tools that you own, and you usually have to bring to your agricultural job?  [multi-select] | 1= Ploughing tools (shovels/hoes/rakes/trowels/pruners/shears/plow/cultivators)  2= Sprayers/pesticide applicators/weeders  3= Irrigation equipment (hoses, sprinklers, drip systems)  4= Harvesting tools (sickles, scythes, knives)  5= Carrying tools (wheelbarrows, bags, basket)  6= Safety gear including gloves and helmet  7= Other, specify: ___ |
| **B.53** Have you attempted to move out of the work you usually do?  [i.e., to get a salaried job in other places such as in manufacturing, processing, etc.] | 1= Yes  2= No |
| **B.54** Reasons you failed to move out of the work you usually do?  [multi-select] | 1= No financial capital  2= Limited education and skills  3= Cannot find a stable job/income in non-agricultural  4= I have someone to take care of  5= I do not want to leave my original place  6= Other, specify: ___ |

CAPI: Ask Module C if B.03==1 | B.03==2

# MODULE C: NON-AGRICULTURAL WORK ACTIVITIES IN THE PAST 12 MONTHS

| **CURRENT RAINY SEASON [mid-May 2023 – late-Oct 2023] NAYON-WASO-WAHGAUNG-TAWTHALIN** | | | | | | | | |
| --- | --- | --- | --- | --- | --- | --- | --- | --- |
| **C.02** How difficult is it to find non-agricultural jobs in the current rainy season? | | | | 1=Very difficult  2=Somewhat difficult  3=No difficulty  4=Don’t do non-agricultural jobs **>> Next season** | | | | |
| **C.03** How did you usually find non-agricultural jobs?  **C.03A The most common one?**  CAPI: preload options selected in C.03A, allow only one of those to be selected. | | | | 1= Through the employer directly  2= Through family/friends/relatives/villagers  3= Through regularly working group member  4= Through co-workers from previous non-agricultural work  5= Through employer/supervisor from previous non-agricultural work  6= Through agent/job agency  7= Other, specify: ____ | | | | |
| **C.04** How many different non-agricultural employers you have been working for in this season? | | | | 1= _____number | | | | |
| **FIRM 1** | | | | | | | | |
| **C.05** Please tell us the details of firm 1 you worked for in this season.  [If respondent worked for more than 1 firm in one season, ask the ONLY ONE that the respondent worked for longest days] – to translate into Myanmar  [Workers no: small firm 10 to 50, Medium 51-100, large 101 and above in accordance with SME development policy in Myanmar] | What task? | Days worked? | Money received | Payment rate? | Total no. of workers? | Relation with owner? | Gender of owner? | Township name |
|  | (#code) |  |  | (#code) |  |  |  | Pre-load |
|  | [multi-select] | __days | __mmk |  | 1. 1 to 5  2. 6 to 10  3. 11 to 20  4. 21 to 30  5. 31 to 40  6. 41 to 50  7. 51 to 100  8. 101 and above  9. none | 1=Relatives  2=Friends/neighbor/villager  3=Other/stranger | 1=Male  2=Female  3=Both | < preload list of S/R; Shan State by North, East and South > + add another S/R (specify 1,2,3) |
| **C.06** How many hours do you usually work per day in each season? | | | | | 1= ____hours__min  2= ____a.m____p.m  3= Do not know | | | |
| **C.07** How many hours of break time do you usually have in a working day (including lunch time)? | | | | | 1= ____hours__min  2= ____a.m____p.m  3= Do not know | | | |
| **C.08** Please tell us the non-wage benefits you receive besides your wages/salaried/crops.  [multi-select] | | | | | 1= Meals  2= No non-wage benefits **>> C.10**  3= Housing/accommodation**>> C.10**  4= Transportation**>> C.10**  5. Refreshments (tea-leaf salad, tea, coffee, soft drinks, etc.,) **>> C.10**  6= Cloths/uniform**>> C.10**  7= Cigarette/betel nut/alcohol/beer**>> C.10**  8= Health insurance/care**>> C.10**  9= Other, specify: ____**>> C.10** | | | |
| **C.09** How often are meals provided per day? | | | | | 1= One meal per day/cash for one meal  2= Two meals per day/cash for two meals  3= Three meals per day/cash three meals  4= No meal provided  5= Other, specify: ____ | | | |
| **C.10** In the case you work with chemicals or assign to risky tasks, do you work usually with protected equipment? | | | | | 1= Yes  2= No (No provided)  3= No (Provided)  4= Not applicable | | | |
| **C.11** Do you usually receive job-related, including safety training? | | | | | 1= Yes  2= No **>> Next season** | | | |
| **C.12** Number of hours/days you receive job-related training? | | | | | 1=___hours  2=___days | | | |

| **C.13** How many days did you work more for other firms in total in this WINTER season? | Average or common amount of wage? | Payment rate? |
| --- | --- | --- |
| __days | __mmk | (#code) |

| **SUMMER SEASON [ mid-Feb 2023 – mid-May 2023] TABAUNG-TAGU-KASON** | | | | | | | | |
| --- | --- | --- | --- | --- | --- | --- | --- | --- |
| **C.02** How difficult it was to find non-agricultural jobs in the last summer season? | | | | 1=Very difficult  2=Somewhat difficult  3=No difficulty  4=Don’t do non-agricultural jobs **>> Next season** | | | | |
| **C.03** How did you usually find non-agricultural jobs? | | | | 1= Through the employer directly  2= Through family/friends/relatives/villagers  3= Through regularly working group member  4= Through co-workers from previous non-agricultural work  5= Through employer/supervisor from previous non-agricultural work  6= Through agent/job agency  7= Other, specify: ____ | | | | |
| **C.04** How many different non-agricultural employers you have been working for in this season? | | | | 1= _____number | | | | |
| **C.04** {$ C.04A} How many of these firms are the same firms in the rainy season? | | | | 1= _____number | | | | |
| **FIRM 1** | | | | | | | | |
| **C.05** Please tell us the details of firm 1 you worked for in this season.  [If respondent worked for more than 1 firm in one season, ask the ONLY ONE that the respondent worked for longest days] translate into Myanmar  [Workers no: small firm 10 to 50, Medium 51-100, large 101 and above in accordance with SME development policy in Myanmar] | What task? | Days worked? | Money received | Payment rate? | Total no. of workers? | Relation with owner? | Gender of owner? | Township name |
|  | (#code) |  |  | (#code) |  |  |  | Pre-load |
|  | {Multi-select] | __days | __mmk |  | 1. 1 to 5  2. 6 to 10  3. 11 to 20  4. 21 to 30  5. 31 to 40  6. 41 to 50  7. 51 to 100  8. 101 and above  9= none | 1=Relatives  2=Friends/neighbor/villager  3=Other/stranger | 1=Male  2=Female  3=Both | < preload list of S/R; Shan State by North, East and South > + another S/R other (specify 1,2,3)  Skip if tasks 5.6.7 |
| **C.06** How many hours do you usually work per day in each season? | | | | | 1= ____hours__min  2= ____a.m____p.m  3= Do not know | | | |
| **C.07** How many hours of break time do you usually have in a working day (including lunch time)? | | | | | 1= ____hours__min  2= ____a.m____p.m  3= Do not know | | | |
| **C.08** Please tell us the non-wage benefits you receive besides your wages/salaried/crops.  [multi-select] | | | | | 1= Meals  2= No non-wage benefits **>> C.10**  3= Housing/accommodation**>> C.10**  4= Transportation**>> C.10**  5. Refreshments (tea-leaf salad, tea, coffee, soft drinks, etc.,) **>> C.10**  6= Cloths/uniform**>> C.10**  7= Cigarette/betel nut/alcohol/beer**>> C.10**  8= Health insurance/care**>> C.10**  9= Other, specify: ____**>> C.10** | | | |
| **C.09** How often are meals provided per day? | | | | | 1= One meal per day/cash for one meal  2= Two meals per day/cash for two meals  3= Three meals per day/cash three meals  4 = No meal provided  5= Other, specify: ____ | | | |
| **C.10** In the case you work with chemicals or assign to risky tasks, do you work usually with protected equipment? | | | | | 1= Yes  2= No (No provided)  3= No (Provided)  4= Not applicable | | | |
| **C.11** Do you usually receive job-related, including safety training? | | | | | 1= Yes  2= No **>> Next season** | | | |
| **C.12** Number of hours/days you receive job-related training? | | | | | 1=___hours  2=___days | | | |

| **C.13** How many days did you work more for other firms in total in this SUMMER season? | Average or common amount of wage? | Payment rate? |
| --- | --- | --- |
| __days | __mmk | (#code) |

| **WINTER SEASON [late-Oct 2022 – mid-Feb 2023] TAZAUNGMON-NATDAW-PYATHOE-TABODWE** | | | | | | | | |
| --- | --- | --- | --- | --- | --- | --- | --- | --- |
| **C.02** How difficult it was to find non-agricultural jobs in the last winter season? | | | | 1=Very difficult  2=Somewhat difficult  3=No difficulty  4=Don’t do non-agricultural jobs **>> Next module D** | | | | |
| **C.03** How did you usually find non-agricultural jobs? | | | | 1= Through the employer directly  2= Through family/friends/relatives/villagers  3= Through regularly working group member  4= Through co-workers from previous non-agricultural work  5= Through employer/supervisor from previous non-agricultural work  6= Through agent/job agency  7= Other, specify: ____ | | | | |
| **C.04** How many different non-agricultural employers you have been working for in this season? | | | | 1= _____number | | | | |
| **C.04** {$C.04A} How many of these firms are the same firms in the summer and rainy (current) season? | | | | 1= _____number | | | | |
| **FIRM 1** | | | | | | | | |
| **C.05** Please tell us the details of firm 1 you worked for in this season.  [If respondent worked for more than 1 firm in one season, ask the ONLY ONE that the respondent worked for longest days] – translate into Myanmar  [Workers no: small firm 10 to 50, Medium 51-100, large 101 and above in accordance with SME development policy in Myanmar] | What task? | Days worked? | Money received | Payment rate? | Total no. of workers? | Relation with owner? | Gender of owner? | Township name |
|  | (#code) |  |  | (#code) |  |  |  |  |
|  | [Multi-select] | __days | __mmk |  | 1. 1 to 5  2. 6 to 10  3. 11 to 20  4. 21 to 30  5. 31 to 40  6. 41 to 50  7. 51 to 100  8. 101 and above  9= None | 1=Relatives  2=Friends/neighbor/villager  3=Other/stranger | 1=Male  2=Female  3=Both | < preload list of S/R; Shan State by North, East and South > + another S/R other (specify 1,2,3)  Skip if tasks 5.6.7 |
| **C.06** How many hours do you usually work per day in each season? | | | | | 1= ____hours__min  2= ____a.m____p.m  3= Do not know | | | |
| **C.07** How many hours of break time do you usually have in a working day (including lunch time)? | | | | | 1= ____hours__min  2= ____a.m____p.m  3= Do not know | | | |
| **C.08** Please tell us the non-wage benefits you receive besides your wages/salaried/crops.  [multi-select] | | | | | 1= Meals  2= No non-wage benefits **>> C.10**  3= Housing/accommodation**>> C.10**  4= Transportation**>> C.10**  5. Refreshments (tea-leaf salad, tea, coffee, soft drinks, etc.,) **>> C.10**  6= Cloths/uniform**>> C.10**  7= Cigarette/betel nut/alcohol/beer**>> C.10**  8= Health insurance/care**>> C.10**  9= Other, specify: ____**>> C.10** | | | |
| **C.09** How often are meals provided per day? | | | | | 1= One meal per day/cash for one meal  2= Two meals per day/cash for two meals  3= Three meals per day/cash three meals  4= no meal provided  5= Other, specify: ____ | | | |
| **C.10** In the case you work with chemicals or assign to risky tasks, do you work usually with protected equipment? | | | | | 1= Yes  2= No (No provided)  3= No (Provided)  4= Not applicable | | | |
| **C.11** Do you usually receive job-related, including safety training? | | | | | 1= Yes  2= No **>> Next module** | | | |
| **C.12** Number of hours/days you receive job-related training? | | | | | 1=___hours  2=___days | | | |

| **C.13** How many days did you work more for other firms in total in this WINTER season? | Average or common amount of wage? | Payment rate? |
| --- | --- | --- |
| __days | __mmk | (#code) |

# MODULE D: ABSENTEEISM AND PRESENTEEISM

Please tell us about your **1-week (7 days)** total work hours. This can be your current work or your most recent work experience while you have a work offer for the whole 1-week.

| **D.01** About how many **hours altogether** did you work in the past 7 days or that week?  [Enumerator: If the respondent provides their daily work schedule including the start and end time, ask about the total number of days worked in a week and the number of hours worked each day. Then, calculate the total hours worked by multiplying the days and hours worked] | 1=________hours  (total days worked x hours per day) |
| --- | --- |
| **D.02** How many **hours** does your employer expect you to work in a typical 7-day week?  [Enumerator: same as C.01] | 1=________hours  (total days worked x hours per day)  9996 = don’t know |
| **D.03** On a scale from 0-10 where 0 is the worst work performance anyone could have at your job and 10 is the performance of a top worker, how would you rate the usual performance of **most** workers in a job similar to yours? | Number 0 to 10 |
| **D.04** Using the same 0 to 10 scale, how would you rate your overall job performance on the days you worked during the **past 1-week (7 days)?** | Number 0 to 10 |

| **D.05** The reasons you did not go to work any day in **the past 1 week (7 days)**?  [multi-select reasons] | 1= Own health  2= Someone else health  3= Physical insecurity  4= Religious and social activities  5= Office appointment  6= Not taken leave  9= Other, specify____ |
| --- | --- |
| **D.06** The reasons you did not go to work any day in **the past 12 months?**  [multi-select reasons] | 1= Own health  2= Someone else health  3= Physical insecurity  4= Religious and social activities  5= Office appointment  6= Not taken leave  9= Other, specify____ |
| **D.07** What do you do when you do not go to work?  [multi-select coping mechanisms] | 1= Household chores  2= Stay at home doing nothing  3= Leave the house and wander around outside  4= Go to drinking alcohol or beer  5= Play cards with friends  6= Do betting for football matches and cockfights  7= Lottering for 2D and 3D  8= Do sporting activities and play games  9= Go to drinking coffee or tea shop  10= Other, specify____ |

**Using 7-day Estimates**

Absolute absenteeism: (4*D.02 – 4*D.01)

Relative absenteeism: (4*D.02 – 4*D.01)/4*D.02

Relative hours of work: D.01/D.02

Absolute presenteeism: 10*D.04

Relative presenteeism: D.04/D.03

# MODULE E: AGRICULTURAL LABOR MARKET CONDITIONS

I now like to know the labor market condition before COVID and Coup 2021 and the current situation.

| **E.01** Did you do agricultural labor before COVID-19? | 1= Yes  2= No **>> F.01** |
| --- | --- |
| **E.02** Compared to before COVID - and now, how would you describe the working days and working hours you have? | 1= Less  2= Same  3= More  4= I do not know |
| **E.03** Compared to before COVID - and now, how would you describe the working opportunity for males? | 1= Less work for male  2= Same  3= More work for male  4= I do not know |
| **E.04** Compared to before COVID - and now, how would you describe the working opportunity for females? | 1= Less work for male  2= Same  3= More work for male  4= I do not know |
| **E.05** Compared to before COVID - and now, how would you describe the safety to travel to the work location? | 1= Lower  2= Higher  3= Same  4= I do not know |
| **E.06** Compared to before COVID - and now, how would you describe travel costs to go to the work location? | 1= Higher cost to travel to work  2= Lower cost to travel to work  3= Same  4= I do not know |
| **E.07** Compared to before COVID - and now, how would you describe the relationship with your employers and co-workers? | 1= Worse  2= Same  3= Better  4= I do not know |
| **E.08** Compared to before COVID - and now, how would you describe stressfulness of going to work? | 1= More stressful  2= Same  3= Less stressful  4= I do not know |

# MODULE F: JOB CHARACTERISTICS

| **F.01** Do you have freedom to decide how you do your task? | 1= No  2= A little  3= Somewhat  4= Very |
| --- | --- |
| **F.02** Do you have freedom to decide when you do your work? | 1= No  2= A little  3= Somewhat  4= Very |
| **F.03** Is the type of work you usually work often requiring you to learn new skills? | 1= No  2= A little  3= A lot  4= Very |
| **F.04** Do you use many of your skills and abilities in the work you usually do? | 1= No  2= A little  3= Alot  4= Very |
| **F.05** Do you usually worry about the future of your work? | 1= No  2= A little worry  3= A lot worry  4= Very worry |
| **F.06** Is the work you usually do complex and difficult? | 1= No  2= A little  3= A lot  4= Very difficult |
| **F.07** Is the task you perform more stressful than it used to be? | 1= No  2= A little stressful  3= A lot stressful  4= Very stressful |
| **F.08** Do you fear that the amount of stress in your work will make you physically ill? | 1= No  2= A little  3= Somewhat  4= Very |
| **F.09** Do you think you will be still doing the same work you usually do in 3 years from now? | 1= No  2= A little  3= Somewhat  4= Very |

# MODULE G: JOB SATISFACTION

I now have some questions about how satisfied or dissatisfied you are with different aspects of your job. If you are not currently employed, these questions refer to your most recent job.

| **G.01** Your total pay | 1= Satisfied  2= Not satisfied |
| --- | --- |
| **G.02** Your job security | 1= Satisfied  2= Not satisfied |
| **G.03** The work itself (what you do) | 1= Satisfied  2= Not satisfied |
| **G.04** The hours you work | 1= Satisfied  2= Not satisfied |
| **G.05** The flexibility available to balance work and non-work commitments | 1= Satisfied  2= Not satisfied |
| **G.06** All thing considered, how satisfied are with your job? | 1= Satisfied  2= Not satisfied |

# MODULE H: LIFE SATISFACTION

| **H.01** The home in which you live | 1= Satisfied  2= Not satisfied |
| --- | --- |
| **H.02** Your employment opportunities | 1= Satisfied  2= Not satisfied |
| **H.03** Your financial situation | 1= Satisfied  2= Not satisfied |
| **H.04** How safe you feel | 1= Satisfied  2= Not satisfied |
| **H.05** Feeling part of your local community | 1= Satisfied  2= Not satisfied |
| **H.06** The amount of time you have | 1= Satisfied  2= Not satisfied |

# MODULE I: HOPKINS SYMPTOMS CHECKLIST (HSCL-10)

Participants will be asked to respond to the following items according to their experience during the previous week (**the past 7 days**). The first 4-items are related to anxiety, and the remaining to depression.

| **Questions** | Answer code  Not at all___(0)  A little ____(1) (1-3 days)  Quite a bit (2)___(4-5 days)  Extremely___(3) (6-7 days)  9997= do not want to say |
| --- | --- |
| **I.01** Suddenly scared for no reasons | Not at all ____ (0)  A little ____ (1)  Quite a bit ____ (2)  Extremely ____ (3) |
| **I.02** Feeling fearful | Not at all ____ (0)  A little ____ (1)  Quite a bit ____ (2)  Extremely ____ (3) |
| **I.03** Faintness, dizziness, or weakness | Not at all ____ (0)  A little ____ (1)  Quite a bit ____ (2)  Extremely ____ (3) |
| **I.04** Feeling tensed or keyed up | Not at all ____ (0)  A little ____ (1)  Quite a bit ____ (2)  Extremely ____ (3) |
| **I.05** Blaming yourself for things | Not at all ____ (0)  A little ____ (1)  Quite a bit ____ (2)  Extremely ____ (3) |
| **I.06** Difficulty in falling asleep or staying asleep | Not at all ____ (0)  A little ____ (1)  Quite a bit ____ (2)  Extremely ____ (3) |
| **I.07** Feeling blue | Not at all ____ (0)  A little ____ (1)  Quite a bit ____ (2)  Extremely ____ (3) |
| **I.08** Feeling of worthlessness | Not at all ____ (0)  A little ____ (1)  Quite a bit ____ (2)  Extremely ____ (3) |
| **I.09** Feeling everything is an effort | Not at all ____ (0)  A little ____ (1)  Quite a bit ____ (2)  Extremely ____ (3) |
| **I.10** Feeing hopeless about future | Not at all ____ (0)  A little ____ (1)  Quite a bit ____ (2)  Extremely ____ (3) |

# MODULE J: WORKPLACE HARASSMENT AND EXPOSURE TO VIOLENCE

| In the past 12 months, has your employer/supervisor/co-worker taken any of the following actions toward you against your will? | |
| --- | --- |
| **J.01** Threatening behavior  [multi-select] | 1= Threatened you  2= Told you that they will harm you if you do not agree to or fulfill their demands  3= No  4= Others, specify: ___ |
| **J.02** Physically  [multi-select] | 1= Hit, slapped, or punched you  2= Cut or stabbed you  3= Intentionally caused you physical harm/tripped you  4= No  5= Others, specify: ___ |
| **J.03** Sexual  [multi-select] | 1= Made remarks about you in a sexual manner  2= Asked or forced you to perform sexual favors  3= Touched you in a sexual manner or in a way  that made you feel uncomfortable or scared.  4= Shown you pictures of sexual activities  5= Others, specify: ___ |

# MODULE K. TRAUMA SCREENING QUESTIONNAIRE (TSQ)

Please consider the following reactions which sometimes occur after a traumatic event. This questionnaire is concerned with your personal reactions to the traumatic event which happened to you. Please indicate (Yes/No) whether or not you have experienced any of the following at least twice in the past week.

| **K.01** Upsetting thoughts or memories about the event that have come into your mind against your will | 1= Yes  2= No |
| --- | --- |
| **K.02** Upsetting dreams about the event | 1= Yes  2= No |
| **K03** Acting or feeling as though the event were happening again | 1= Yes  2= No |
| **K.04** Feeling upset by reminders of the event | 1= Yes  2= No |
| **K.05** Bodily reactions (such as fast heartbeat, stomach churning, sweatiness, dizziness) when reminded of the event | 1= Yes  2= No |
| **K.06** Difficulty falling or staying asleep | 1= Yes  2= No |
| **K.07** Irritability or outbursts of anger | 1= Yes  2= No |
| **K.08** Difficulty concentrating | 1= Yes  2= No |
| **K.09** Heightened awareness of potential dangers to yourself and others | 1= Yes  2= No |
| **K.10** Being jumpy or being startled at something unexpected | 1= Yes  2= No |

If you have answered yes to 6 or more questions you are encouraged to consider whether you think that some counseling support may be of benefit in helping you to lower your on-going reactions to the traumatic event.

| K.11 Do you want a contact for the counselling service? | 1= Yes  2= No |
| --- | --- |

**MODULE L: ACCESS TO INFORMATION**

| **L.01** How do you usually get news about politics, economics and social issues and more?  [multi-select] | 1= Facebook  2= Telegram  3= Viber  4= Signal  5= Newspaper (printed)  6= Radio  7= Television  9= Mouth of words (skip L02)  8= Others, specify: ___ |
| --- | --- |
| **L02** How many hours do you spend to read news? | 1= Less day an hour per day  2= 1-2 hours per day  3= More than 2 hours per day  4= A few times in a week  5= Others, specify: ___ |
| **L.03** How many hours do you spend on social media per day? | 1= Less day an hour per day  2= 1-2 hours per day  3= More than 2 hours per day  4= A few times in a week  5= Others, specify: ___ |

| **Gregorian months** | **Myanmar calendar months** | **Season** |
| --- | --- | --- |
| Feb-Mar | Tabaung | Summer |
| Mar-Apr | Tagu |  |
| Apr-May | Kason |  |
| May-June | Nayon | Rainy |
| June-July | Waso |  |
| July-August | Wahgaung |  |
| August-Sep | Tawthalin |  |
| Sep-Oct | Thadingyut |  |
| Oct-Nov | Tazaungmon | Winter |
| Nov-Dec | Natdaw |  |
| Dec-Jan | Pyathoe |  |
| Jan-Feb | Tabodwe |  |

| **Agricultural tasks Code Unit (on farm)** | **Other Income-Generating Tasks Code Unit (out of farm)** |
| --- | --- |
| 1= Ploughing by hand or animals (e.g., tools, cattle)  2= Seedling/broadcasting/transplanting  3= Pesticide/Herbicide/Fertilizer/Manure application  4= Weeding/Cleaning farms/pulling grass/cutting grass including, livestock feed for others  5= Irrigation (e.g., fixing irrigation system, watering, etc.)  6= Harvesting manually (e.g., threshing, rubber tapping, picking fruits, etc.)  7= Machinery driver (e.g., small tractor, 1-2/2-4-wheels, power tiller, harvester/combined harvester)  8= Machinery assistant  9= Aggregating crops, sorting, and loading on truck (only within the farm)  10= Others specify: _____ | 1= Construction and building (brick mason, carpenter, road pavement, etc.)  2= Driving (truck, bus, taxi, motorbike-carrier, rickshaw puller)  3= Loading and unloading work at transportation hubs, including bus or train stations or warehouse  4= Working for others (i.e., as a helper including, household chores, laundry, cleaning and selling so on)  5= Others specify: _____ |
| **Payment rate** |  |
| 1= Per hour  2= Per half day  3= Per day  4= Per week  5= Per month  6= Per task  7= Per Acre  8= Per Hectare  9= Lumpsum amount  10= Other, specify: ____ |  |

| Code Unit | Weight unit for other bags |
| --- | --- |
| 1.    Pyi  2.    Viss  3.    Standard basket (16 pyi)  4.    100 standard basket (1600 pyi)  5.    Pone (8 pyi)  6.    Pone (7.5 pyi)  7.    Pone (6 pyi)  8.    Rakhine basket (6 pyi)  9.    Lan  10.  Bag (30.75 viss)  11.  Bag (108 lb)  12.  Bag (1.5 basket/ 24 pyi)  13.  Other bag (Specify it in the standard unit)>>weight unit  14.  Pounds  15.  Tons | 1.    viss  2.    kg  3.    pound  4.    basket  5.    pyi |

| Code : Crop | Code 6: Crop | Code 6: Crop |
| --- | --- | --- |
| ----------------------TYPE 1 -------------------------  Grains  Rice................................................................ 119  Paddy................................................................ 1  Maize ............................................................... 2  Sweetcorn........................................................ 96  Wheat............................................................... 3  Sorghum........................................................... 4  Millet ............................................................... 5  Barley ............................................................... 6  Pulses, oilseeds, beans  Groundnut........................................................ 7  Soybean............................................................ 8  Sesame............................................................. 9  Sunflower......................................................... 10  Niger................................................................. 11  Green gram...................................................... 12  Black gram........................................................ 13  Chickpea........................................................... 14  Lab Lab bean.................................................... 15  Butter bean...................................................... 16  Rice bean.......................................................... 17  Pigeon pea........................................................ 20  Cowpea/black-eyed pea ……………………………...117  Kidney bean ………………………………………………..118  Tubers/Root crop  Taro.................................................................. 21  Potato............................................................... 22  Arrow root........................................................ 23  Cassava............................................................. 24  Elephant foot yum............................................ 25  Sweet potato………………………………….26  Fruits  Banana ............................................................. 27  Mango ............................................................. 28  Apple................................................................ 29  Watermelon..................................................... 30  Muskmelon...................................................... 31  Pineapple.......................................................... 32  Strawberry........................................................ 33  Avocado ........................................................... 34  Dragon Fruit..................................................... 35  Pear.................................................................. 36  Jackfruit............................................................ 37  Durian............................................................... 38  Papaya.............................................................. 39 | ----------------------TYPE 2 (cont’d)---------------  Orange.............................................................. 40  Citron................................................................ 41  Pomelo............................................................. 42  Guava............................................................... 43  Grapefruit......................................................... 44  Grapes.............................................................. 45  Rambutan......................................................... 46  Lychee.............................................................. 47  Dragon eye....................................................... 48  Persimmon....................................................... 49  Passion............................................................. 50  Mulberry........................................................... 51  Jujube............................................................... 52  Plum................................................................. 53  Custard apple................................................... 54  Nuts  Walnut.............................................................. 55  Chestnut........................................................... 56  Almond............................................................. 57  Cashew nut ...................................................... 58  Macadamia....................................................... 59  Other trees  Sugarcane ........................................................ 60  Coffee............................................................... 61  Tea.................................................................... 62  Coconut............................................................ 63  Betel Nut ......................................................... 64  Betel Leaves..................................................... 65  Cheroot (dried)................................................. 66  Dog fruit (Djenkol bean)................................... 67  Rubber.............................................................. 68  Teak.................................................................. 69  Eucalyptus ....................................................... 70  Other woods..................................................... 71  Bamboo............................................................ 72  Vegetables  Water cress...................................................... 73  Roselle ............................................................. 74  Luffa.................................................................. 75  Shallot............................................................... 76  Lettuce/Celtuce................................................ 77  Onion................................................................ 78 | ----------------------TYPE 2 (cont’d) -------------  Vegetables  Cabbage............................................................ 79  Cauliflower ...................................................... 80  Mustard/Kailan/Pakchoi.................................. 81  Mushroom........................................................ 82  Radish............................................................... 83  Carrot............................................................... 84  Okra.................................................................. 85  Tomato ............................................................ 86  Eggplant ........................................................... 87  Long bean......................................................... 88  Green bean....................................................... 89  Cucumber......................................................... 90  Gourd/Chayote................................................. 91  Bitter gourd...................................................... 92  Pumpkin........................................................... 93  Ash pumpkin.................................................... 94  Winged bean.................................................... 95  Drumstick tree.................................................. 97  Lemon/Lime..................................................... 98  Chili (fresh)....................................................... 99  Chili (dried)....................................................... 100  Tamarind.......................................................... 101  Ornamental flowers  Roses................................................................ 102  Lily.................................................................... 103  Eugenia............................................................. 104  Chrysanthemum............................................... 105  Other ornamental flowers (specify)................. 106  Spices/Herbs  Ginger .............................................................. 107  Turmeric........................................................... 108  Garlic................................................................ 109  Pepper.............................................................. 110  Cardamom........................................................ 111  Bay tree............................................................ 112  Gamone (Medical Herb)................................... 113  Other Crops  Poppy................................................................ 114  Hemp................................................................ 115  Sericulture (silk)............................................... 116  Other (Specify)................................................. 9999  Tobacco (dried)................................................ 18  Cotton............................................................... 19 |
