# Supplementary material for: Conflict, mental health, and labor productivity: evidence from hired farm workers in Myanmar
Source: BMC Glob Public Health. 2026 Jul 29;4:72. doi: 10.1186/s44263-026-00307-5 (PMC13421825; doi:10.1186/s44263-026-00307-5)
Supplement: Supplementary file 3 — Supplementary Material 3: The Stata Code for Analysis. Stata do-file used for statistical analyses. [file 44263_2026_307_MOESM3_ESM.docx]

Conflict, mental health, and labor productivity: Evidence from hired farm workers in Myanmar

**Supplementary Results**

Table of Contents

[1. Descriptive results 2](#_Toc227669237)

[2. Sensitivity analyses 7](#_Toc227669238)

[2.1 Treating the CSI as a continuous variable and alternative conflict proxy (fatalities) 7](#_Toc227669239)

[2.2 Conflict timeframe at 6 and 12 months 8](#_Toc227669240)

[2.3 Conflict Severity Index (CSI) within a 50 km radius 11](#_Toc227669241)

[2.4 Treating absenteeism as continuous, performance as a binary, and mental health as a continuous 12](#_Toc227669242)

[2.5 Alternative mental health measure: Hopkins Symptoms Checklist (HSCL-10) 13](#_Toc227669243)

[2.6 Health differences 14](#_Toc227669244)

[3. Gendered differences 15](#_Toc227669245)

[References 16](#_Toc227669246)

# **1. Descriptive results**

| Table S1. Share of townships affected by conflict by state/region/territory | | |
| --- | --- | --- |
| State/region/territory names | Share of townships affected by conflict | Total number of townships |
| 1. Nay Pyi Taw (Union territory) | 0.000 | 8 |
| 2. Ayeyarwady Region | 0.000 | 26 |
| 3. Yangon Region | 0.000 | 16 |
| 4. Shan State | 0.200 | 30 |
| 5. Bago Region | 0.269 | 26 |
| 6. Mandalay Region | 0.292 | 24 |
| 7. Magway Region | 0.304 | 23 |
| 8. Mon State | 0.333 | 9 |
| 9. Rakhine State | 0.375 | 16 |
| 10. Chin State | 0.600 | 5 |
| 11. Kayin State | 0.667 | 6 |
| 12. Kayah State | 0.667 | 3 |
| 13. Tanintharyi Region | 0.800 | 10 |
| 14. Kachin State | 0.800 | 10 |
| 15. Sagaing Region | 0.900 | 30 |
| Average share (State/region/territory) | 0.414 | 16.133 |
| *Note:* Conflict is measured as a binary variable, taking the value 1 if the Conflict Severity Index (CSI) is greater than 0 and 0 if CSI equals 0. To gain additional insights into within variation, we regress the treatment variable on location (7 states, 7 regions, and 1 union territory) fixed effects and find an R-squared of 0.450 and a residuals variance of 0.130 for the conflict variable, also suggesting meaningful variation. | | |

| Table S2. Descriptive statistics by trauma status | | | |
| --- | --- | --- | --- |
|  | (1) | (2) | (3) |
| Variables | Trauma | No Trauma | p-value |
| Dependent variables (outcomes) |  |  |  |
| Absenteeism (0/1) | 0.347 | 0.222 | 0.000 |
|  | (0.027) | (0.012) |  |
| Work performance (0-10 scale) | 6.987 | 7.299 | 0.036 |
|  | (0.136) | (0.067) |  |
| Treatment variable |  |  |  |
| Conflict Severity Index (0/1) | 0.505 | 0.356 | 0.000 |
|  | (0.028) | (0.014) |  |
| Control variables |  |  |  |
| Male (0/1) | 0.350 | 0.402 | 0.094 |
|  | (0.027) | (0.014) |  |
| Age (in years) | 36.583 | 36.387 | 0.782 |
|  | (0.599) | (0.326) |  |
| Work experience (in years) | 10.786 | 10.428 | 0.548 |
|  | (0.533) | (0.720) |  |
| Married (0/1) | 0.731 | 0.719 | 0.661 |
|  | (0.025) | (0.013) |  |
| Primary education (0/1) | 0.259 | 0.285 | 0.371 |
|  | (0.025) | (0.013) |  |
| Employer is a relative (0/1) | 0.178 | 0.186 | 0.753 |
|  | (0.022) | (0.011) |  |
| Employer is a friend (0/1) | 0.505 | 0.528 | 0.467 |
|  | (0.028) | (0.014) |  |
| Employer is a stranger (0/1) | 0.201 | 0.146 | 0.018 |
|  | (0.023) | (0.010) |  |
| Lives in rural area (0/1) | 0.935 | 0.934 | 0.930 |
|  | (0.014) | (0.007) |  |
| Observations (N) | 309 | 1,195 |  |
| *Note:* Standard errors in parentheses. Column (3) displays the p-value of a two-sample t-test, comparing respondents experiencing trauma (Column 1) and those who are not (Column 2). The measurement of all variables is described in the methods section of the main paper. | | | |

| Table S3. Descriptive statistics by trauma status, among the sup-sample of respondents exposed to conflict | | | |
| --- | --- | --- | --- |
|  | (1) | (2) | (3) |
| Variables | Trauma | No trauma | p-value |
| Dependent variables (outcomes) |  |  |  |
| Absenteeism (0/1) | 0.372 | 0.252 | 0.005 |
|  | (0.039) | (0.021) |  |
| Work performance (0-10 scale) | 6.673 | 7.125 | 0.041 |
|  | (0.176) | (0.117) |  |
| Control variables |  |  |  |
| Male (0/1) | 0.353 | 0.398 | 0.323 |
|  | (0.038) | (0.024) |  |
| Age (in years) | 36.179 | 35.605 | 0.572 |
|  | (0.864) | (0.528) |  |
| Work experience (in years) | 10.500 | 9.981 | 0.551 |
|  | (0.751) | (0.449) |  |
| Married (0/1) | 0.686 | 0.729 | 0.303 |
|  | (0.037) | (0.022) |  |
| Primary education (0/1) | 0.173 | 0.249 | 0.052 |
|  | (0.030) | (0.021) |  |
| Employer is a relative (0/1) | 0.192 | 0.188 | 0.912 |
|  | (0.032) | (0.019) |  |
| Employer is a friend (0/1) | 0.462 | 0.565 | 0.027 |
|  | (0.040) | (0.024) |  |
| Employer is a stranger (0/1) | 0.212 | 0.106 | 0.001 |
|  | (0.033) | (0.015) |  |
| Lives in rural areas (0/1) | 0.910 | 0.936 | 0.275 |
|  | (0.023) | (0.012) |  |
| Observations (N) | 156 | 425 |  |
| *Note:* Standard errors in parentheses. Column (3) displays the p-value of a two-sample t-test, comparing respondents experiencing trauma (Column 1) and those who are not (Column 2). The measurement of all variables is described in the methods section of the main paper. | | | |

| Panel A |
| --- |
| 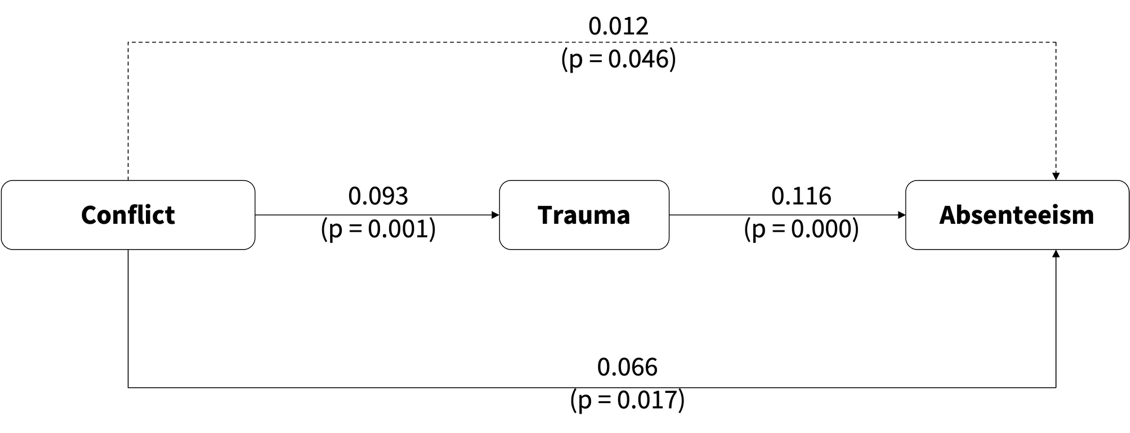 |
| Panel B |
| 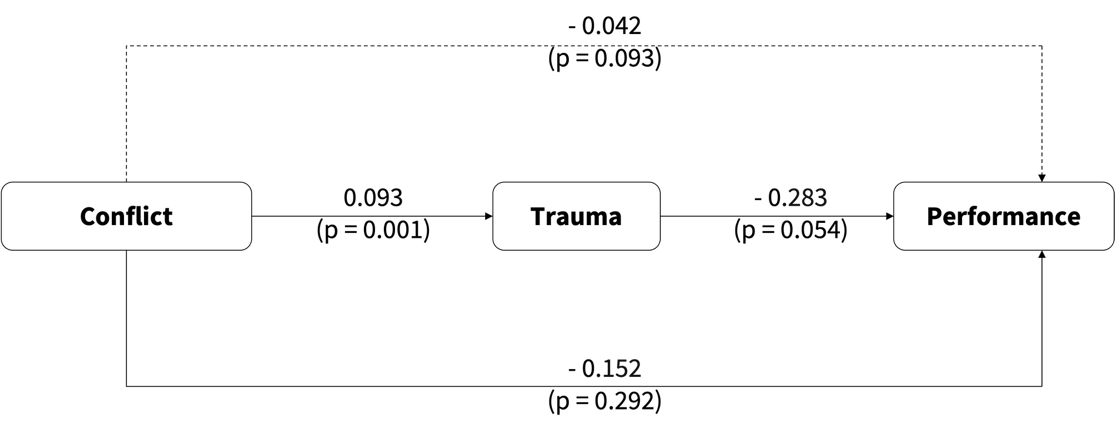 |
| Fig. S1. The direct and indirect associations between conflict, mental health, and labor productivity.  *Note:* This diagram visually illustrates the key results on how conflict is associated with labor productivity outcomes (i.e., absenteeism—Panel A—and work performance—Panel B), both directly and indirectly through its relationship with mental health problems (trauma). The arrows indicate the direction of the relationships among the three main variables. The straight arrow (bottom) indicates the direct association, while the dotted arrow (above) indicates indirect associations. The total associations are the sum of the direct and indirect associations. The total association between conflict and absenteeism is 0.075 (p = 0.027), and for work performance it is −0.194 (p = 0.170). |

| Table S4. Direct and indirect associations between conflict, mental health, and absenteeism (left) and work performance (right) | | | | | | | | | | | | |
| --- | --- | --- | --- | --- | --- | --- | --- | --- | --- | --- | --- | --- |
|  |  | (1) |  | (2) |  | (3) |  | (1) |  | (2) |  | (3) |
|  | Absenteeism | | | | | | Work performance | | | | | |
|  | Beta | p-value | Beta | p-value | Beta | p-value | Beta | p-value | Beta | p-value | Beta | p-value |
| Direct association | 0.047 | 0.038 | 0.046 | 0.046 | 0.063 | 0.022 | -0.330 | 0.010 | -0.304 | 0.016 | -0.150 | 0.302 |
|  | (0.023) |  | (0.023) |  | (0.028) |  | (0.129) |  | (0.126) |  | (0.145) |  |
| Indirect association | 0.013 | 0.025 | 0.012 | 0.031 | 0.013 | 0.035 | -0.046 | 0.091 | -0.042 | 0.108 | -0.044 | 0.085 |
|  | (0.006) |  | (0.006) |  | (0.006) |  | (0.027) |  | (0.026) |  | (0.026) |  |
| Total association | 0.060 | 0.007 | 0.058 | 0.010 | 0.076 | 0.004 | -0.377 | 0.002 | -0.346 | 0.004 | -0.194 | 0.169 |
|  | (0.022) |  | (0.022) |  | (0.026) |  | (0.124) |  | (0.121) |  | (0.141) |  |
| *Controls* | *No* |  | *Yes* |  | *No* |  | *No* |  | *Yes* |  | *No* |  |
| *Location fixed effects* | *No* |  | *No* |  | *Yes* |  | *No* |  | *No* |  | *Yes* |  |
| Observations (N) |  |  | 14,84 |  |  |  |  |  | 1,504 |  |  |  |
| *Note:* Beta = coefficient. Standard errors in parentheses, clustered at the township level. Location fixed effects are included at the second administrative level (7 states, 7 regions, and 1 union territory). Conflict is measured as a binary variable, taking the value 1 if the Conflict Severity Index (CSI) is greater than 0 and 0 if CSI equals 0. Mental health is a binary indicator equal to 1 if the trauma score is ≥ 5, and 0 otherwise. Absenteeism is also measured as a binary indicator equal to 1 if any hours were missed in the past 7 days, and 0 otherwise. Work performance is measured as a continuous variable (0-10). | | | | | | | | | | | | |

# **2. Sensitivity analyses**

## **2.1 Treating the CSI as a continuous variable and alternative conflict proxy (fatalities)**

| Table S5. Direct and indirect associations between conflict, mental health, and absenteeism (left) and work performance (right) | | | | |
| --- | --- | --- | --- | --- |
|  | Absenteeism | | Work performance | |
|  | Beta | p-value | Beta | p-value |
| Direct association | 0.052 | 0.165 | -0.036 | 0.848 |
|  | (0.038) |  | (0.187) |  |
| Indirect association | 0.014 | 0.150 | -0.047 | 0.208 |
|  | (0.010) |  | (0.038) |  |
| Total association | 0.067 | 0.065 | -0.083 | 0.668 |
|  | (0.036) |  | (0.194) |  |
| *Controls* | *Yes* |  | *Yes* |  |
| *Location fixed effects* | *Yes* |  | *Yes* |  |
| Observations (N) | 1,484 |  | 1,504 |  |
| *Note:* Beta = coefficient. Standard errors in parentheses, clustered at the township level. Location fixed effects are included at the second administrative level (7 states, 7 regions, and 1 union territory). Conflict is measured as a continuous variable (0-4). Mental health is a binary indicator equal to 1 if the trauma score is ≥ 5, and 0 otherwise. Absenteeism is also measured as a binary indicator equal to 1 if any hours were missed in the past 7 days, and 0 otherwise. Work performance is measured as a continuous variable (0-10). | | | | |

| Table S6. Direct and indirect associations between conflict, mental health, and absenteeism (left) and work performance (right) | | | | |
| --- | --- | --- | --- | --- |
|  | Absenteeism | | Work performance | |
|  | Beta | p-value | Beta | p-value |
| Direct association | 0.051 | 0.093 | -0.126 | 0.446 |
|  | (0.030) |  | (0.165) |  |
| Indirect association | 0.007 | 0.076 | -0.019 | 0.141 |
|  | (0.004) |  | (0.013) |  |
| Total association | 0.058 | 0.057 | -0.145 | 0.379 |
|  | (0.030) |  | (0.164) |  |
| *Controls* | *Yes* |  | *Yes* |  |
| *Location fixed effects* | *Yes* |  | *Yes* |  |
| Observations (N) | 1,484 |  | 1,504 |  |
| *Note:* Beta = coefficient. Standard errors in parentheses, clustered at the township level. Location fixed effects are included at the second administrative level (7 states, 7 regions, and 1 union territory). Conflict is measured as a binary variable, taking the value 1 if there is any fatality and 0 if there is no fatality. Mental health is a binary indicator equal to 1 if the trauma score is ≥ 5, and 0 otherwise. Absenteeism is also measured as a binary indicator equal to 1 if any hours were missed in the past 7 days, and 0 otherwise. Work performance is measured as a continuous variable (0-10). | | | | |

## **2.2 Conflict timeframe at 6 and 12 months**

|  |  |
| --- | --- |
| Fig. S2. Conflict severity (proxied using the Conflict Severity Index - CSI) over 6 months.  *Note:* The CSI timeframe is between 28 May and 28 November 2023. The thin dark lines represent the borders of 330 townships, at which the CSI is measured. The thick dark lines represent the borders of 7 states, 7 regions, and 1 union territory. Panel A represents the Conflict Severity Index (CSI), which ranges from 0 to 4, while Panel B shows the dummy variable indicating either no conflict (CSI = 0) or conflict (CSI = 1-4). | |

| Table S7. Direct and indirect associations between conflict, mental health, and absenteeism (left) and work performance (right) | | | | |
| --- | --- | --- | --- | --- |
|  | Absenteeism | | Work performance | |
|  | Beta | p-value | Beta | p-value |
| Direct association | 0.056 | 0.040 | 0.021 | 0.883 |
|  | (0.027) |  | (0.140) |  |
| Indirect association | 0.009 | 0.051 | -0.025 | 0.151 |
|  | (0.004) |  | (0.018) |  |
| Total association | 0.064 | 0.017 | -0.004 | 0.975 |
|  | (0.027) |  | (0.140) |  |
| *Controls* | *Yes* |  | *Yes* |  |
| *Location fixed effects* | *Yes* |  | *Yes* |  |
| Observations (N) | 1,484 |  | 1,504 |  |
| *Note:* Beta = coefficient. Standard errors in parentheses, clustered at the township level. Location fixed effects are included at the second administrative level (7 states, 7 regions, and 1 union territory). Conflict is measured as a binary variable, taking the value 1 if the Conflict Severity Index (CSI) is greater than 0 and 0 if CSI equals 0. Mental health is a binary indicator equal to 1 if the trauma score is ≥ 5, and 0 otherwise. Absenteeism is also measured as a binary indicator equal to 1 if any hours were missed in the past 7 days, and 0 otherwise. Work performance is measured as a continuous variable (0-10). | | | | |

|  |  |
| --- | --- |
| Fig. S3. Conflict severity (proxied using the Conflict Severity Index - CSI) over 12 months*.*  *Note:* The CSI timeframe is between 28 November 2022 and 28 November 2023. The thin dark lines represent the borders of 330 townships, at which the CSI is measured. The thick dark lines represent the borders of 7 states, 7 regions, and 1 union territory. Panel A represents the Conflict Severity Index (CSI), which ranges from 0 to 4, while Panel B shows the dummy variable indicating either no conflict (CSI = 0) or conflict (CSI = 1-4). | |

| Table S8. Direct and indirect associations between conflict, mental health, and absenteeism (left) and work performance (right) | | | | |
| --- | --- | --- | --- | --- |
|  | Absenteeism | | Work performance | |
|  | Beta | p-value | Beta | p-value |
| Direct association | 0.050 | 0.070 | -0.031 | 0.823 |
|  | (0.028) |  | (0.141) |  |
| Indirect association | 0.007 | 0.106 | -0.019 | 0.204 |
|  | (0.004) |  | (0.015) |  |
| Total association | 0.057 | 0.039 | -0.050 | 0.721 |
|  | (0.028) |  | (0.141) |  |
| *Controls* | *Yes* |  | *Yes* |  |
| *Location fixed effects* | *Yes* |  | *Yes* |  |
| Observations (N) | 1,484 |  | 1,504 |  |
| *Note:* Beta = coefficient. Standard errors in parentheses, clustered at the township level. Location fixed effects are included at the second administrative level (7 states, 7 regions, and 1 union territory). Conflict is measured as a binary variable, taking the value 1 if the Conflict Severity Index (CSI) is greater than 0 and 0 if CSI equals 0. Mental health is a binary indicator equal to 1 if the trauma score is ≥ 5, and 0 otherwise. Absenteeism is also measured as a binary indicator equal to 1 if any hours were missed in the past 7 days, and 0 otherwise. Work performance is measured as a continuous variable (0-10). | | | | |

## **2.3 Conflict Severity Index (CSI) within a 50 km radius**

| Table S9. Direct and indirect associations between conflict, mental health, and absenteeism (left) and work performance (right) | | | | |
| --- | --- | --- | --- | --- |
|  | Absenteeism | | Work performance | |
|  | Beta | p-value | Beta | p-value |
| Direct association | 0.131 | 0.792 | -0.164 | 0.479 |
|  | (0.050) |  | (0.232) |  |
| Indirect association | 0.010 | 0.250 | -0.039 | 0.271 |
|  | (0.009) |  | (0.036) |  |
| Total association | 0.023 | 0.650 | -0.203 | 0.384 |
|  | (0.051) |  | (0.234) |  |
| *Controls* | *Yes* |  | *Yes* |  |
| *Location fixed effects* | *Yes* |  | *Yes* |  |
| Observations (N) | 1,484 |  | 1,504 |  |
| *Note:* Beta = coefficient. Standard errors in parentheses, clustered at the 50 km buffer level. Conflict is measured as a binary variable, taking the value 1 if the Conflict Severity Index (CSI) is greater than 0 and 0 if CSI equals 0. Mental health is a binary indicator equal to 1 if the trauma score is ≥ 5, and 0 otherwise. Absenteeism is also measured as a binary indicator equal to 1 if any hours were missed in the past 7 days, and 0 otherwise. Work performance is measured as a continuous variable (0-10). | | | | |

## **2.4 Treating absenteeism as continuous, performance as a binary, and mental health as a continuous**

| Table S10. Direct and indirect associations between conflict, mental health, and absenteeism (left) and work performance (right) | | | | |
| --- | --- | --- | --- | --- |
|  | Absenteeism | | Work performance | |
|  | Beta | p-value | Beta | p-value |
| Direct association | 0.147 | 0.081 | -0.030 | 0.341 |
|  | (0.084) |  | (0.031) |  |
| Indirect association | 0.025 | 0.112 | -0.009 | 0.089 |
|  | (0.016) |  | (0.006) |  |
| Total association | 0.172 | 0.033 | -0.039 | 0.209 |
|  | (0.081) |  | (0.031) |  |
| *Controls* | *Yes* |  | *Yes* |  |
| *Location fixed effects* | *Yes* |  | *Yes* |  |
| Observations (N) | 1,484 |  | 1,504 |  |
| *Note:* Beta = coefficient. Standard errors in parentheses, clustered at the township level. Location fixed effects are included at the second administrative level (7 states, 7 regions, and 1 union territory). Conflict is measured as a binary variable, taking the value 1 if the Conflict Severity Index (CSI) is greater than 0 and 0 if CSI equals 0. Trauma is measured as a continuous variable (0-10). Absenteeism is treated as a continuous log variable. Work performance is treated as a dummy variable. The cut-off for work performance is set at 7 or above (coded as 1) and below 7 (coded as 0), as 7 represents the average work performance on the 0-10 scale. | | | | |

| Table S11. Direct and indirect associations between conflict, mental health, and absenteeism (left) and work performance (right) | | | | |
| --- | --- | --- | --- | --- |
|  | Absenteeism | | Work performance | |
|  | Beta | p-value | Beta | p-value |
| Direct association | 0.067 | 0.017 | -0.169 | 0.246 |
|  | (0.028) |  | (0.146) |  |
| Indirect association | 0.013 | 0.023 | -0.028 | 0.274 |
|  | (0.006) |  | (0.026) |  |
| Total association | 0.079 | 0.003 | -0.198 | 0.164 |
|  | (0.027) |  | (0.142) |  |
| *Controls* | *Yes* |  | *Yes* |  |
| *Location fixed effects* | *Yes* |  | *Yes* |  |
| Observations (N) | 1,484 |  | 1,504 |  |
| *Note:* Beta = coefficient. Standard errors in parentheses, clustered at the township level. Location fixed effects are included at the second administrative level (7 states, 7 regions, and 1 union territory). Conflict is measured as a binary variable, taking the value 1 if the Conflict Severity Index (CSI) is greater than 0 and 0 if CSI equals 0. Trauma is measured as a continuous variable (0-10). Absenteeism is measured as a binary indicator equal to 1 if any hours were missed in the past 7 days, and 0 otherwise. Work performance is treated as a continuous variable (0-10). | | | | |

## **2.5 Alternative mental health measure: Hopkins Symptoms Checklist (HSCL-10)**

We use the Hopkins Symptoms Checklist (HSCL-10) to measure workers’ mental distress[1–3]. The HSCL-10 consists of 10 items, which assess anxiety (the first 4 items) and depression (the remaining 6 items) symptoms in the past seven days. For each item, respondents indicate on a 0–4 scale (0 = no experience, 1 = 1-3 days experience, 2 = 2-4 days experience, 3 = 6-7 days experience). The internal consistencies and reliability for HSCL-10 (Cronbach $\alpha$= 0.86) are similar to the previous study of the Myanmar urban population (Cronbach $\alpha$= 0.85) and Pakistan (Cronbach $\alpha$= 0.86) population-based studies[1,3]. To calculate the mean score, we divided the sum of all item scores by 10. Using a standard cut-off, we define mental distress as a dummy variable that equals one if the mean score equals or exceeds 1.85, and zero otherwise[4]. Our descriptive statistics indicate that the prevalence of mental distress among farm workers in Myanmar is 17%, which is 3 percentage points lower than the prevalence of trauma (20%). And respondents with complex mental health problems are 12%.

| Table S12. Direct and indirect associations between conflict, mental health, and absenteeism (left) and work performance (right) | | | | |
| --- | --- | --- | --- | --- |
|  | Absenteeism | | Work performance | |
|  | Beta | p-value | Beta | p-value |
| Direct association | 0.073 | 0.007 | -0.218 | 0.134 |
|  | (0.027) |  | (0.146) |  |
| Indirect association | 0.005 | 0.186 | 0.006 | 0.582 |
|  | (0.004) |  | (0.012) |  |
| Total association | 0.078 | 0.003 | -0.212 | 0.139 |
|  | (0.027) |  | (0.143) |  |
| *Controls* | *Yes* |  | *Yes* |  |
| *Location fixed effects* | *Yes* |  | *Yes* |  |
| Observations (N) | 1,484 |  | 1,504 |  |
| *Note:* Beta = coefficient. Standard errors in parentheses, clustered at the township level. Location fixed effects are included at the second administrative level (7 states, 7 regions, and 1 union territory). Conflict is measured as a binary variable, taking the value 1 if the Conflict Severity Index (CSI) is greater than 0 and 0 if CSI equals 0. Mental health (mental distress) is measured as a binary variable, taking the value 1 if the HSCL-10 is greater than or equal to 8 or 1.25, otherwise 0. Absenteeism is also measured as a binary indicator equal to 1 if any hours were missed in the past 7 days, and 0 otherwise. Work performance is measured as a continuous variable (0-10). | | | | |

## **2.6 Health differences**

| Table S13. Direct and indirect associations between conflict, mental health, and absenteeism (left) and work performance (right) | | | | |
| --- | --- | --- | --- | --- |
|  | Absenteeism | | Work performance | |
|  | Beta | p-value | Beta | p-value |
| Direct association | 0.145 | 0.001 | -0.085 | 0.684 |
|  | (0.043) |  | (0.210) |  |
| Indirect association | 0.012 | 0.237 | -0.030 | 0.466 |
|  | (0.010) |  | (0.041) |  |
| Total association | 0.156 | 0.000 | -0.116 | 0.574 |
|  | (0.042) |  | (0.206) |  |
| *Controls* | *Yes* |  | *Yes* |  |
| *Location fixed effects* | *Yes* |  | *Yes* |  |
| Observations (N) | 638 |  | 649 |  |
| *Note:* Beta = coefficient. Standard errors in parentheses, clustered at the township level. Location fixed effects are included at the second administrative level (7 states, 7 regions, and 1 union territory). Conflict is measured as a binary variable, taking the value 1 if the Conflict Severity Index (CSI) is greater than 0 and 0 if CSI equals 0. Mental health is a binary indicator equal to 1 if the trauma score is ≥ 5, and 0 otherwise. Absenteeism is also measured as a binary indicator equal to 1 if any hours were missed in the past 7 days, and 0 otherwise. Work performance is measured as a continuous variable (0-10). | | | | |

# **3. Gendered differences**

| Table S14. Descriptive statistics comparing women versus men in conflict areas | | | |
| --- | --- | --- | --- |
| Variables | Men | Women | p-value |
| Dependent variables (outcomes) |  |  |  |
| Absenteeism (0/1) | 0.269 | 0.294 | 0.523 |
|  | (0.030) | (0.024) |  |
| Work performance (0-10 scale) | 7.116 | 6.933 | 0.363 |
|  | (0.156) | (0.126) |  |
|  |  |  |  |
| Mediating variable |  |  |  |
| Trauma (0/1) | 0.246 | 0.283 | 0.363 |
|  | (0.029) | (0.024) |  |
| Control variables |  |  |  |
| Age (in years) | 35.723 | 35.782 | 0.950 |
|  | (0.762) | (0.556) |  |
| Work experience (in years) | 9.960 | 10.221 | 0.741 |
|  | (0.634) | (0.485) |  |
| Married (0/1) | 0.741 | 0.703 | 0.323 |
|  | (0.029) | (0.024) |  |
| Primary education (0/1) | 0.205 | 0.244 | 0.285 |
|  | (0.027) | (0.023) |  |
| Employer is a relative (0/1) | 0.183 | 0.193 | 0.760 |
|  | (0.026) | (0.021) |  |
| Employer is a friend (0/1) | 0.509 | 0.555 | 0.283 |
|  | (0.033) | (0.026) |  |
| Employer is a stranger (0/1) | 0.165 | 0.115 | 0.084 |
|  | (0.025) | (0.017) |  |
| Lives in rural areas (0/1) | 0.165 | 0.941 | 0.163 |
|  | (0.019) | (0.012) |  |
| Observations (N) | 224 | 357 |  |
| *Note:* Standard errors in parentheses. Column (3) displays the p-value of a two-sample t-test, comparing Men (Column 1) and Women (Column 2). The measurement of all variables is described in the methods section of the main paper. | | | |

# **References**

1. Aye WT, Lien L, Stigum H, Win HH, Oo T, Bjertness E. The prevalence of mental distress and the association with education: a cross-sectional study of 18-49-year-old citizens of Yangon Region, Myanmar. BMC Public Health. 2020 Dec;20(1):94. doi:10.1186/s12889-020-8209-8

2. Schmalbach B, Zenger M, Tibubos AN, Kliem S, Petrowski K, Brähler E. Psychometric properties of two brief versions of the Hopkins Symptom Checklist: HSCL-5 and HSCL-10. Assessment. 2021 Mar;28(2):617–31. doi:10.1177/1073191119860910

3. Syed HR, Zachrisson HD, Dalgard OS, Dalen I, Ahlberg N. Concordance between Hopkins Symptom Checklist (HSCL-10) and Pakistan Anxiety and Depression Questionnaire (PADQ), in a rural self-motivated population in Pakistan. BMC Psychiatry. 2008 Dec;8(1):59. doi:10.1186/1471-244X-8-59

4. Strand BH, Dalgard OS, Tambs K, Rognerud M. Measuring the mental health status of the Norwegian population: a comparison of the instruments SCL-25, SCL-10, SCL-5 and MHI-5 (SF-36). Nordic journal of psychiatry. 2003;57(2):113–8.
